# Business oriented EU human cell and tissue product legislation will adversely impact Member States’ health care systems

**DOI:** 10.1007/s10561-013-9397-6

**Published:** 2013-09-20

**Authors:** Jean-Paul Pirnay, Alain Vanderkelen, Daniel De Vos, Jean-Pierre Draye, Thomas Rose, Carl Ceulemans, Nadine Ectors, Isabelle Huys, Serge Jennes, Gilbert Verbeken

**Affiliations:** 1 Human Cell and Tissue Banks, Laboratory for Molecular and Cellular Technology (LabMCT), Queen Astrid Military Hospital, Brussels, Belgium; 2 Department of Behavioural Sciences, Royal Military Academy, Brussels, Belgium; 3 Tissue Banks, University Hospitals Leuven, KU Leuven, Leuven, Belgium; 4 Department of Pharmaceutical and Pharmacological Sciences, Centre for Pharmaceutical Care and Pharmacoeconomics, KU Leuven, Leuven, Belgium; 5 Center for Intellectual Property Rights, KU Leuven, Leuven, Belgium; 6 Burn Wound Centre, Queen Astrid Military Hospital, Brussels, Belgium

**Keywords:** Cell and tissue banking, Tissue engineering, Advanced therapy medicinal product, Regulation, European Union, Public health

## Abstract

The transplantation of conventional human cell and tissue grafts, such as heart valve replacements and skin for severely burnt patients, has saved many lives over the last decades. The late eighties saw the emergence of tissue engineering with the focus on the development of biological substitutes that restore or improve tissue function. In the nineties, at the height of the tissue engineering hype, industry incited policymakers to create a European regulatory environment, which would facilitate the emergence of a strong single market for tissue engineered products and their starting materials (human cells and tissues). In this paper we analyze the elaboration process of this new European Union (EU) human cell and tissue product regulatory regime—i.e. the EU Cell and Tissue Directives (EUCTDs) and the Advanced Therapy Medicinal Product (ATMP) Regulation and evaluate its impact on Member States’ health care systems. We demonstrate that the successful lobbying on key areas of regulatory and policy processes by industry, in congruence with Europe’s risk aversion and urge to promote growth and jobs, led to excessively business oriented legislation. Expensive industry oriented requirements were introduced and contentious social and ethical issues were excluded. We found indications that this new EU safety and health legislation will adversely impact Member States’ health care systems; since 30 December 2012 (the end of the ATMP transitional period) there is a clear threat to the sustainability of some lifesaving and established ATMPs that were provided by public health institutions and small and medium-sized enterprises under the frame of the EUCTDs. In the light of the current economic crisis it is not clear how social security systems will cope with the inflation of costs associated with this new regulatory regime and how priorities will be set with regard to reimbursement decisions. We argue that the ATMP Regulation should urgently be revised to focus on delivering affordable therapies to all who are in need of them and this without necessarily going to the market. The most rapid and elegant way to achieve this would be for the European Commission to publish an interpretative document on “placing on the market of ATMPs,” which keeps tailor-made and niche ATMPs outside of the scope of the medicinal product regulation.

## Introduction

For decades, the transplantation of human cells, tissues, and cellular and tissue-based products (HCT/Ps) like heart valve replacements for patients with heart insufficiency and skin for severely burnt patients has saved many lives, restoring essential functions where no real alternatives of comparable effectiveness exist. Recently, within the emerging field of regenerative medicine, tissue engineering became a much-hyped component. Examples of applications for human tissue engineered products (hTEPs) are treatment possibilities for common conditions including chronic wounds and bone diseases or injuries, or niche applications such as severe burns. The most appealing perspective for this sub-theme of HCT/Ps, however, is to be able to regenerate whole organs (e.g. heart, liver, kidney or trachea) and hence overcome the shortage of donor organs for transplantation.

### Cell and tissue directives

In 2004, the European Commission (EC), the main originator of legislation in the European Union (EU) political process, issued the EU Cell and Tissue Directives (EUCTDs), which consist of three Directives: the parent Directive 2004/23/EC (European Union 2004), which provides the framework legislation and two technical directives, 2006/17/EC and 2006/86/EC (European Union 2006a, b), which provide the detailed requirements of the parent Directive. They were designed to assure harmonized and high standards of quality and safety for the donation, procurement, testing, processing, preservation, storage and distribution of human cells and tissues for human applications, to facilitate their cross-border movements and to ensure availability in the EU. Tissues and cells intended to be used for industrially manufactured products and medical devices are covered by the EUCTDs only as far as donation, procurement and testing are concerned. These Directives introduced requirements for human cell and tissue establishments, which necessitated extensive reorganizations and investments, but are today perceived as overall positive.

### Advanced therapy medicinal product (ATMP) regulation

In 2005, the EC decided to classify all “innovative, regenerative therapies which combine aspects of medicine, cell biology, science and engineering for the purpose of regenerating, repairing or replacing damaged tissues or cells,” such as hTEPs, under the heading “ATMP.” This implies that from that moment hTEPs were considered as human medicinal products. An ATMP “contains or consists of cells or tissues that have either been subject to *substantial manipulation* or that are not intended to be used for the same essential function(s) in the recipient as in the donor and is presented as having properties for treating or preventing disease in patients.” Expansion by culturing is currently by default considered to be a substantial manipulation. Since 2008, these ATMPs are regulated in Regulation 1394/2007/EC (European Union 2007), which does not derogate from the EUCTDs, but supplements them with additional requirements such as production according to good manufacturing practice (GMP) and compliance with marketing authorization requirements and post-marketing pharmacovigilance rules. The ATMP Regulation was designed to allow free movement of ATMPs within the EU market, better patient access to ATMPs, the highest level of health protection for patients, EU competitiveness in a key biotechnology area and growth of an emerging industry.

### Back to the future

In the summer of 2012, Belgian keratinocyte banks received a letter from the Belgian national competent authority (NCA) for medicines. In this letter the public cell banks were notified that their “products,” human keratinocytes for the treatment of burns and chronic skin wounds, falls under the definition of an ATMP and that the administration of this product to patients as it is currently performed—i.e. exclusively under the frame of the EUCTDs—is not allowed beyond 30 December 2012.

On the one hand, it was flattering to learn that keratinocyte banks, since their foundation in the eighties, had been delivering grafts, which today are considered to be *advanced* products. On the other hand it was confusing to learn that the cell banks would not be allowed to continue the administration of grafts to patients under the EUCTDs, especially since these keratinocyte grafts had been applied on more than 1,000 severely burnt patients (De Corte et al. 2012) and periodic inspections by the NCAs had not revealed significant quality or safety issues.

### Placing on the market?

Because the ATMP Regulation is a *lex specialis* inside the medicinal product Directive 2001/83/EC (European Union 2001a), it addresses all ATMPs falling within the global scope of Community legislation on medicinal products, i.e. “medicinal products for human use intended to be *placed on the market* in Member States and either prepared industrially or manufactured by a method involving an industrial process.” Remarkably, there is no definition of “placed on the market” in the field of medicinal products in the European Law. The field of medical devices, however, provides a definition in Directive 93/42/EEC43 (European Union 1993) and in 2010 the EC published an interpretative document on “placing on the market of medical devices” (European Union 2010a; Klumb 2011). Accordingly, for EU manufacturers a product is considered placed on the market when the product is transferred from the stage of manufacture with the intention of distribution or use on the Community market. This transfer can consist in a physical hand-over and/or be based on a legal transaction. It can relate to the ownership, the possession or any other right transferred from the manufacturer to a distributor or to the end user. A transfer of a product is considered to have taken place, e.g. when it is sold, leased, given as a gift, rent out or hired. One would think that non-anonymized cells and tissues for autologous use remain the propriety of the donor and are thus not transferred or placed on the market. But, within the European Medicines Agency (EMA), the Committee for Advanced Therapies (CAT), which issues scientific recommendations determining whether or not a referred product falls within the definition of an ATMP, already considered several autologous cell therapies to be ATMPs (EMA/CAT 2012a). In its argumentation to the NCA, the keratinocyte bank of the Queen Astrid Military Hospital argued that the application of their keratinocyte grafts exclusively on their patients, at no charge for the patient, would not qualify as “placing on the market” and that their “product” would thus not fall within the global scope of Community legislation on medicinal products. Conform to the hierarchy in EU Law, if the medicinal product Directive is not applicable, nor is the ATMP Regulation and as a consequence the keratinocytes should not be classified as an ATMP. This view was backed by a specialized law firm (Bredin Prat 2012), but not by the authorities so far.

### The commercialization of altruism

The troubling question is: “how did HCT/Ps originating from altruistic donations and delivered by not-for-profit public cell and tissue banks become commercial medicinal products?”

In the EU—as is the case in the United Sates (US) as well—it is illegal to buy and sell human cells and tissues. The principle that it is not permissible for the human body or its parts to give rise to financial gain was established in Article 21 of the 1997 *Council of Europe Convention of Human Rights and Biomedicine* (Council of Europe 1997). Nevertheless, in practice, human cells and tissues are “sold” across borders worldwide, as it is not illegal to compensate hospitals, coroners and morgues for reasonable costs and charge “reasonable fees” for the processing rather than the direct purchase of human cells and tissues. The HCT/P transplantation field, which used to be dominated by altruistic hospital-based tissue banks, is increasingly occupied and steered by tissue brokers and (stock exchange listed) corporate tissue establishments in pursuit of profits, particularly in the US where the market value of transplants from one body—not including solid organs—was estimated at $230,000 in the year 2000 (Collins 2001). Expectations regarding the potential markets that hTEPs (or ATMPs in general) could cover are even higher. In the EU, a significant part of tissue establishments are still operating on a strictly not-for-profit basis, although it must be said that some of them have been set up by private industries, particularly for the supply of starting materials for the production of hTEPs (Table 1—1.4). International brokers supply human organs, cells and tissues, obtained in low-income countries without self-sufficiency, basically located in Africa, Asia, Eastern Europe and South America, to the powerful industry in human tissues (Council of Europe 2009). But, the emerging global commercialization of human cells and tissues is fraught with dangers (Pirnay et al. 2012a). Ethical and safety issues involving illegal and fraudulent activities (IFAs) (Collins 2001) and legal excessive profit making activities (LEPRAs; Pirnay et al. 2012a) soon emerged and questioned the adequacy of the regulatory framework that governed the HCT/P transplantation field. Critics of markets in body parts state that these practices violate a fundamental ethical norm that the body should not be treated either as a property or as a commodity (Council of Europe 1997). On the one hand, there are clear indications that EU policymakers wish to avoid the commercialization and commodification of human body parts. On the other hand, the recent EU HCT/P regulatory framework (EUCTDs and ATMP Regulation) is business-oriented in its origin: it allows for-profit tissue establishments in all kinds and facilitates the development of a uniform and global HCT/P market, but at the same time it is not able to deal with the controversial market-driven practices that raise deep ethical issues (Council of Europe 2009). Today, the commercialization of human cells and tissues is a fact, as are the malpractices in the field, and the EU is maintaining a stand of tolerance.

**Table 1:** Textual extracts (statements, evaluations and perspectives) from reports of studies commissioned by the EC or industry (listed according to publication date)

1. Opinion of the European Group on Ethics in Science and New Technologies to the European Commission: Ethical aspects of human tissue banking (EGE 1998)
1.1. Wherever tissues are removed from human beings, and possibly transplanted into other human beings, the activities involved in the collection and use of such tissues are subject to ethical requirements intended to safeguard respect for human beings, their dignity and autonomy, and for the common good
1.2. All Member States of the EU adhere to the principle that donations of human tissues must be free, following the example of blood, and this rules out any payment to the donor. However, the donor may receive compensation for the constraints associated with tissue removal (e.g. travel expenses, loss of earnings, etc.). Some parties maintain that for the sake of fairness, when the tissues become even indirectly a source of profit, donors should be paid. Furthermore, donor’s remuneration might increase the supply of tissues. So far, however, the arguments in favour of the altruistic nature of tissue donation (like organ donation) have prevailed. They are based on a regard for solidarity. Also they are inspired by the desire to avoid the human person being regarded as an object (a source of organs and tissues). Another argument in favour of free donations is to avoid all risk of exploitation of the most underprivileged who might be led, in doubtful conditions of health, to donate tissue exclusively or primarily for financial reasons
1.3. The issue of the commercialization of human tissues, which have been processed and prepared for therapeutic purposes, is even more controversial. For some, tissue banks must be operated only by non-profit-making bodies, as the tissues have originally been obtained free of charge in a spirit of altruism. For others hold that the processing and conversion of tissues involve costs, which they believe justify their commercial sale, in the same way as blood derivatives. The commercialization of human tissues has the added advantage, according to its proponents, of encouraging industry to invest in areas, which will result in greater availability of tissues on the market. This argument is most often advanced with regard to ‘engineered’ tissues requiring sophisticated industrial processing techniques
1.4. Currently, although no surveys of tissue banks in Europe have been carried out, it seems that most of the banks are non-profit; nevertheless some of them have been set up by private industries, particularly for the production of engineered tissues
1.5. In principle, tissue bank activities should be reserved to public health institutions or non-profit-making organizations. In such case, this means that the delivery price of the tissues only covers the bank’s expenses relating to the tissues in question. Nevertheless, given the current state of development of the sector, it is difficult to exclude tissue-banking activities by commercial organizations, such as large private laboratories. This is particularly true where human tissues are used as a basis for “engineered” products requiring the use of sophisticated medical techniques. Tissue banks set up by industry should be subject to the same licensing and monitoring requirements as non-commercial operators
2. Working Group’s written reports from the “Meeting on the Therapeutic Use of Human Organs and Tissues” held in Porto from 14 to 16 June 2000 (Loty et al. 2000)
2.1. The Amsterdam Treaty clarifies the horizontal nature of Community Health policy implying the obligation to ensure the protection of Public Health in all EU policies. Specifically the paragraph 4 of Article 152 states that the Council must adopt “… measures setting high standards of quality and safety of organs and substances of human origin…”
2.2. Scope should consider the 2 documents: Opinion of the European Group on Ethics in Science and New Technologies to the European Commission: Ethical Aspects of Human Tissue Banking, 21 July 1998 Safety and quality assurance for Organs, Tissues and Cells, version 8, 26 April 2000 from the European Council
2.3. The survey of existing regulation in European Member States showed several oppositions on ethical aspects, many similarities on safety aspects, but also a lack of regulation in many countries
2.4. Considering the increasing number of tissues and cells exchanged between countries, there is an unanimity of all experts to express the urgent need for a regulation on tissues and cells. Support from all participants for European Directives on human tissue. General principles need to be addressed in first instance in a directive. Annexes of this document should then provide more detail and address certain issues (e.g. safety plus quality aspects)
2.5. Recognition of the need for a recognized system of unified regulatory controls, including specific regulations, and inspection for these activities for each type of tissues or cells
2.6. Regulation of structures and activities could be at national or central European level (e.g. traditional tissues at national level, innovative tissues by central European level)
2.7. Issues such as Vigilance and Traceability should be addressed more fully
2.8. The Organs Working Group agreed that legal initiatives should address the shortage of organs and tissues and that no new legislation should be enacted that limits the availability of living and cadaveric donors
3. Proposal for a Directive of the European Parliament and of the Council on setting standards of quality and safety for the donation, procurement, testing, processing, storage, and distribution of human tissues and cells (European Union 2002a)
3.1. The legal basis for this proposal is Article 152 of the Treaty, in particular (4)(a), which requires the European Parliament and the Council to adopt measures that set high standards of quality and safety of substances of human origin
3.2. The measures set out in this proposed Directive incorporate requirements for the procurement, testing, processing, storage, and distribution of tissues and cells of human origin intended for application in the human body. They do not prevent Member States from maintaining or introducing more stringent protective measures, in conformity with the Treaty, and do not affect national provisions on the donation or medical use of tissues and cells of human origin
3.3. In contrast to existing European Community procedures concerning the approximation of laws, regulations and administrative provisions relating to proprietary medicinal products, this proposed Directive does not have as its primary objective the placing on the market of tissues and cells of human origin
3.4. Autologous cells used for medicinal products require a completely different regulatory approach and therefore are completely excluded from this Directive. Tissues and cells used as an autologous graft (tissues removed and transplanted back to the same person), within the same surgical procedure and without being subjected to any banking process, are also excluded from this proposal. The quality and safety considerations associated with this process are completely different
3.5. As a matter of principle, tissue and cell transplantation programs should be founded on the philosophy of voluntary and unpaid donation, anonymity of both donor and recipient, benevolence of the donor and encouragement of the absence of profit by establishments involved in tissue and cell transplantation services
3.6. The tissue and cell establishments directly concerned by the provision of this proposal vary from tissue banks, to health centers where procurement is carried out, to third parties, which can be responsible for some step of the process. The proposal will have indirect implications on the tissue engineering products industry. The requirements of this Directive may increase the cost of starting materials used by business
3.7. No specific provision is envisaged for small and medium sized firms in this proposal
4. EuropaBio’s proposals on DG Sanco’s proposed Directive regarding quality and safety of tissues and cells presented during a public hearing in the European Parliament on 29 January 2003 (EuropaBio 2002)
4.1. Industry to be accredited as Tissue Bank. Industry has the expertise in development of innovative products. Treaty 152: Conflict occurs when a Member State does not grant accreditation as tissue bank to industry!
4.2. No need for industry to operate on 24-h basis
4.3. Contracted third party of a tissue bank should be allowed to distribute
4.4. Harmonize the scope of Directive for autologous and allogeneic cells used for industrially manufactured products for medical use
4.5. Harmonized European regulations (considering Treaty 152) enabling small & medium-size companies investing in development of cell & tissue engineered products
5. DG JRC-IPTS study report: Human Tissue Engineered Products—Today‘s Markets and Future Prospects (Bock et al. 2003)
5.1. More sophisticated and novel hTEPs (e.g. tissue-engineered intervertebral discs, larger bone substitutes, tissue-engineered heart valves) might become available in the foreseeable future
5.2. Initiatives like LIFE (Living Implants from Engineering, USA) in 1999 promised to be able to tissue engineer a human heart within 10 years. The time scale for a lab-grown heart has subsequently been extended to about 25 years by the founder of LIFE (Zandonella 2003), which may still be significantly over-optimistic
5.3. The European *market* is characterized by young, small, research-based and technology-oriented companies, most of them SMEs with less than 50 employees
5.4. In addition to companies, also tissue banks and hospital laboratories produce hTEPs. However, there are only limited data available for Germany, the UK, and France on the scope and extent of their tissue engineering activities. It seems that currently hospitals carry out research or produce fairly simple, autologous hTEPs for in-house treatments. Tissue banks consider tissue engineering as a future strategic option, but do not yet produce any hTEPs. Activities presently seem to be limited to a few institutions per country
5.5. Tissue-engineered products differ in many ways from medical devices and pharmaceuticals. For that reason they are not appropriately covered by current European legislation. The EC is approaching this issue via new European legislation. A directive on standards for quality and safety of human tissues and cells is already in the decision process of the European institutions, a regulation covering human tissue-engineered products is currently being developed
5.6. Due to difficulties with reimbursement by European health insurance schemes of treatments based on tissue-engineered skin products companies target increasingly the ‘self-payer’ patients segment, such as aesthetic surgery
5.7. Because of a lack of strong evidence for superiority of tissue engineering treatments the cost-effectiveness for burn treatment favours the conventional treatment
6. DG JRC-IPTS study report: Human Tissue Engineered products: Potential Socio-economic Impacts of a New European Regulatory Framework for Authorisation, Supervision and Vigilance (Bock et al. 2005)
6.1. It seems that currently hospitals carry out research or produce fairly simple, autologous hTEPs for in-house treatments. Tissue banks consider tissue engineering as a future strategic option, but do not yet produce any hTEPs
6.2. It can be expected that the current trend of concentration due to adaptation to national and European standards (e.g. Directive 2004/23/EC) will continue. Adapting to and compliance with the regulation could tie up resources that might otherwise be available for investment in R&D. This is felt to be particularly likely in the case of SMEs. As well as delaying the launch of hTEPs and limiting the range a given company develops and produces, this could tip the scales in favour of larger firms better able to target pan-European markets. This could then lead to market consolidation in the form of takeovers or product licensing
6.3. Providers of equipment or GMP grade ancillary reagents could see increased sales in the short term as hTEP manufacturers adapt
6.4. Downstream players such as doctors, patients and insurers might face higher product prices as companies seek to recoup their increased compliance costs
6.5. Reimbursement policies are particularly significant. Currently, hTEPs are much more expensive than conventional treatment options and cost-effectiveness data are scarce. Product prices may rise initially as a result of higher regulatory compliance costs, but increased competition and economies of scale could eventually drive hTEP prices down
7. Eucomed Proposal for a Regulation of the European Parliament and of the Council on Advanced Therapies and amending Regulation (EC) No 726/2004 (Eucomed 2004)
7.1 NOTE: The point under (a) would not ensure equal access for patients to high level safety, quality and effective hTEP This Regulation shall not apply to: a) Any advanced therapy medicinal product that is prepared by a qualified and licensed professional, such as a pharmacist, physician, or trained and certified biologist, on an exceptional basis, in order to comply with a medical prescription for an individual patient; the product must be prepared in full at the site of treatment of the patient, and without using standardised or patented processes;
8. Eucomed position paper on the proposal for a Community Regulatory Framework on Advanced Therapies of 04 May 2005 (Eucomed 2005)
8.1. We would like to join the other Trade Federations in offering our help, with our experts, for the elaboration of the numerous guidance documents, at all levels, requested by the Regulation. We believe that the experience gained by Eucomed members in the Medical Devices field in providing loyal support to regulators in the elaboration of sound and balanced guidance is a wealth to be fully exploited also in this area
8.2. We believe that it is paramount to ensure that the regulatory regime takes into account the speed of innovation in this sector, the technology used (much more engineering oriented rather than pure pharmaceutical oriented) and the needs of the patients, who cannot wait too long for access to the health products they need, in certain cases, to survive. This, of course, does not imply that the level of controls should be less than rigorous, but it also has to be appropriate in order to achieve the end objective: timely, effective, safe and quality patient care
8.3. It must also be noted that the times and fees for approval are extremely critical to encourage (or discourage) not only SMEs, which represent the large majority of manufacturers of these products, but also big Corporations to invest in this promising branch of medical technology
8.4. We do not have philosophical objections to the fact that the EMEA (European Medicines Agency) will deal with hTEPs, on the contrary, we believe that a centralized approach may help in creating a favorable environment for the development of this innovative technology
8.5. We believe that the European Union should be a level playing field for those researching, designing and manufacturing hTEPs, but over all, we believe that patients should be entitled to have access to hTEPs based on the highest safety, quality and efficacy standards. This cannot be reached if different rules apply depending on the nature of the business of the manufacturer. For this reason, we oppose the creation of special rules for “one-off, non-industrially manufactured” hTEPs
9. Impact Assessment. Annex to the ‘Proposal for a Regulation on Advanced Therapy Medicinal Products’ (European Union 2005a)
9.1. At the Commission’s request the EGE has examined the potential ethical issues raised by the introduction of a common framework for TEPs. These issues were analysed in the light of previous opinions of the Group as well as other reference documents such as the European Charter of Fundamental Rights and the Convention for the protection of human rights and dignity of the human being with regard to the application of biology and medicine: Convention on human rights and biomedicine (“Oviedo” Convention)
9.2. While the other advanced therapy products have been regulated as medicinal products for many years within the Community, tissue-engineered products currently lie outside of any EU legislative framework. This leads to divergent national approaches as to their legal classification and authorisation, which impair the free movement of these products, hinder patients’ access to innovative therapies, and ultimately affect the EU competitiveness in this key biotechnology area
9.3. A few hospitals and tissue banks have developed, or are planning to develop, large-scale tissue engineering manufacturing facilities. They can, therefore, be regarded as competitors to tissue engineering companies. This is true in particular for institutions, which intend to rely on industrial processes and to make their products available to patients and/or to other operators on their home market or beyond national borders
9.4. In terms of future market developments, the outcome of potential competition between tissue engineering companies and hospitals/tissue banks is open due to the often public, non-profit character of the latter. The fixed production costs are considered to be similar for both types of actors. However, hospitals and tissue banks normally have less marketing costs and do not calculate profit margins. On the other hand, tissue-engineering companies might be able to exploit economies of scale due to a national or international orientation and have more incentives for a rationalised production process
9.5. Competition between tissue engineering companies and health institutions is expected to remain limited in the short to medium term. Many hospitals do not intend to develop important facilities for producing a large number of TEPs. Their main interest is in providing optimal treatments to their own patients, on a non-industrial scale. This will be done either through cooperation with tissue engineering companies, or through the development of tailored tissue engineering treatments
9.6. “Placing on the market”: some respondents considered the proposed definition of “placing on the market” as improper because it does not cover products manufactured and used in the same facility (in-house use, for instance in hospitals). They stressed that there is no reason why such products should not be subject to the same rules as tissue engineered products manufactured by industrial operators. A large majority of stakeholders were of the opinion that hospitals, tissue banks and other local actors should be subject to the same rules as enterprises
9.7. On the other hand, other stakeholders from the ‘Healthcare professionals’ and ‘Research’ category stressed that the exclusion was too narrow, that the concept of ‘industrial manufacturing process’ may be too vague and that hospitals and university/research environments should not be imposed unnecessary regulatory overburdens such as marketing authorisation requirements
9.8. Upstream players. Providers of cells and tissues will have to comply—if they haven’t already—with the provisions laid down in Directive 2004/23/EC as far as donation, procurement and testing of cells and tissues are concerned. There will be no additional requirements on the basis of the proposed Regulation
9.9. Detailed guidelines. As for gene and somatic cell therapy products, detailed technical guidance would be drawn up for tissue-engineered products through guidelines, drawn up either by the EMEA or by the Commission. The fact that expertise is still scarce in this fast-growing, fast-evolving area highlights the importance of extensive and thorough consultation with all interested parties, in particular the industry, for the drafting of these guidelines
9.10. Directive 2004/23/EC provides for quality and safety standards for the donation, procurement, testing, processing, preservation, storage and distribution of human tissues and cells. However, it does not address efficacy aspects, does not lay down rules for the evaluation and marketing authorisation of tissue/cell-based manufactured products, and also does not cover products based on animal cells
9.11. Public budgets will be affected by the proposed Regulation through the costs incurred by the mandatory manufacturing authorisation and the post-authorisation surveillance for TEPs
9.12. Lastly, there may also be a potential indirect impact on public expenditure through pricing and reimbursement of advanced therapy products. This ‘pricing and reimbursement’ aspect falls under the responsibility of Member States
9.13. Donor information: a few stakeholders requested that the donor be informed of the usage made of the tissue which they provide as source material
10. Report of EuropaBio’s Industry Hearing, Tissue Engineering and Advanced Therapies (Geesink 2005)
10.1. Many stakeholders wondered about the meaning of the term engineered, arguing how this concept should be more precisely defined, to prevent borderline issues with other cell based products. In response to these concerns the Commission drafted a more precise definition of engineering, following the FDA approach in what are considered non-substantial manipulations for a tissue engineered product. *Nicolas Rossignol from DG Enterprise on an issue that was raised during the public consultation*
10.2. A final point to consider concerns the explicit exclusion for certain products produced and used in hospitals. “We don’t want to impose a too heavy regulatory burden or requirements on hospitals, but on the other hand of course we need to ensure a level playing field for the different actors. And that’s a balance that I’m sure will be heavily discussed at the Council.” *Nicolas Rossignol*
10.3. The UK houses many small spin off companies and specialist research hospitals, and tissue engineering R&D takes place on a very small and developmental scale. “It is very strongly iterative and characterised by the gradual emergence of efficacy. In many ways what is happening is at the borderline between procedure and product. We also feel strongly that it is important to recognise the gradation of risk that will vary widely according to the specific product.” The issue of hospital production is another concern on the UK list. This is of particular importance to the UK, and obviously this bears on the issue of regulatory impact of hospital production on a tiny scale, and potentially it will not be realistic to put together dossiers for such a tiny number of products per year. Technical requirements have been questioned as well. “Clearly these need to be risk based, and fully proportionate, to reflect the characteristics of the individual product.” *Richard Woodfield from the UK Medicines and Healthcare Product Regulatory Agency (MHRA)*
10.4. There is overall support for the Commission’s proposals from the EMEA Committee for Medicinal Products for Human Use (the CHMP). However, they still have concerns that centralised licensing may be difficult for hospital-produced products
10.5. “The point was made that the regulatory framework is a necessary, but not sufficient, step to make tissue engineered treatments available to patients: Member States have to be prepared to pay to make them available to those in need.” *Peter Liese, Member of the European Parliament (MEP), Chairman of the European People’s Party (EPP) working group on bioethics, member of the Committee on the Environment, Public Health and Safety and rapporteur for the EUCTDs*
10.6. Liese stresses how this Regulation “has always been asked for by industry” but also that “we have to draft this legislation in such a way that those companies that are covered by medical devices are happy with it.”
10.7. Liese also stresses that the issue of hospital based services should be addressed, on which stakeholders hold very different positions. “There should be similar rules for the public sector, for hospitals, and for industry. But of course you have to draw the borderline.”
10.8. The fact that the Sanco Directive (the EUCTD) is a Directive and the fact that the new Advanced Therapies Regulation is a Regulation may cause a conflict in certain areas. For example this fact has led some Member States to implement rules that go much further than the Sanco Directive. For example let’s assume the Advanced Therapies Regulation is there and a company wants to market a product and so receives a marketing authorisation. However in certain countries the company can’t access primary material, because they would have to be registered as a tissue bank, which is not required by the Sanco Directive
10.9. All healthcare products are aimed at the well being of patients, but not all healthcare products are made in the same way, belong to the same industry or to the same technology. “And among other things, human tissue engineered products are not medicinal products. They cannot be.” In other words the proposed regulatory framework, now mostly based on pharmaceutical and medical device legislation, needs adaptation for tissue-engineered products. For example Directive 726/2004 needs to be adapted with respect to the application of the GMP and the application of the Directive on clinical investigation. *Dario Pirovano, regulatory affairs director of EUCOMED*
10.10. SMEs support the centralised procedure approach and welcome the provisions that have already been implemented such as the product designation meetings at the EMEA, the reduction of fees, and the support for translation in all official languages, which all really support SMEs. *Nancy Veulemans, regulatory consultant for TiGenix, an SME trying to file a cartilage product*
10.11. Furthermore Veulemans discusses the compatibility of the Regulation with the Sanco Directive. This Directive states that access to primary material should be guaranteed and that manufacturers are allowed to settle themselves as tissue establishment. “We have already heard that this Directive is based on art 152 of the Treaty, and that Member States may add extra measures on top of that. We have heard from cases where member States are already adding these provisions. And that would be one question: if the Member States are and will always be allowed to add extra measures under article 152, and have this already in place, how can this disappear again? So, the de facto situation of this development is that we have potentially 25 different systems for access to primary material for manufacturers before the process can start. But, some countries are at this moment denying companies to establish themselves as tissue establishment. Others require contracts, strong QA and audit in place with each individual hospital and doctor, at the hospital’s premises, in order to be allowed to do a ‘procurement’. So this means that access to primary material is not guaranteed and patients are denied or will be denied promising treatments.”
10.12. Niese explains how the regulatory environment for this product in European countries ranged from unregulated to transplant to medical devices to pharmaceutical legislation “For a big company, that always has to operate on a global scale, this means a no go.” But also some level of harmonisation for reimbursement is needed, or at least agreement on the principles for evaluation and reimbursement. *Detlef Niese, head of external relations clinical development and medical affairs at Novartis the large pharma company that worked with US biotech company Organogenesis on the development and marketing of Apligraf, the first tissue engineered skin product commercially available*
10.13. The next question is for Rossignol again, and refers to the fact that more and more doctors use materials and equipment for peri-operative processing of cells, and the question is whether this will be kept outside any regulation, especially given that these processes are similar to those performed by industry, which is subject to regulation. Rossignol answers this is a question of scope, and the balance between the level playing field on the one hand, which is needed by industry, versus tissue banks, and on the other hand the regulatory burden or requirements put on stakeholders, and in particular hospitals. “And I’m afraid we will have to wait for the Council discussion on this. At the end of the day this discussion is also mirroring the real political debate behind this, which is the respected competence between Member States and the Community.”
10.14. Legislation at European level is needed, the Commission proposal is welcomed. We sincerely expect quick and positive progress, as the patients deserve it, the patients need it, the patients are expecting it. *Johan Vanhemelrijkck, EuropaBio’s Secretary General*
10.15. We heard from the SMEs, and inherent to what was said by the SMEs there were three words very important: cost, cost, cost. So be very careful not to kill the SME or the product with demands that go over the capacity.” *Johan Vanhemelrijkck*
10.16. Conclusion. There is a clear need for tissue engineering to be regulated if companies are to be able to license new products and for patients across Europe to receive the benefits of these. The needs of SMEs, which make up an important part of the bio-medical sector clearly are being addressed, and hopefully will be dealt with fairly under the new legislation
11. Eucomed Advanced Therapy Medicinal Products Backgrounder (Eucomed 2008)
11.1. Patients should be assured that the treatments they receive are safe, are of high quality, and perform as intended, no matter who prepares the treatment. The text needs to be amended to ensure that this is the case. Currently the proposal is worded in such a way that hospitals might be able to avoid complying with the provisions of the regulation, whereas industrial manufacturers of similar products would bear the obligations of compliance
11.2. There are already products available to patients on a national basis in certain member states, e.g. Germany. These have been authorised for use by the national authorisation systems. Patients should be assured that the treatment they are currently receiving will not be taken away from them during the process of implementing this new legislation. The current proposal foresees a transition period of just 2 years for these existing products to comply with the new provisions. Companies are going to want to comply with the new regulation because of the support and incentives it offers. Forcing them to do so in an unrealistic timetable will not be good for patients

In this paper we attempt to explain how this paradox came about. We demonstrate that industry’s disproportionate influence on key areas of regulatory and policy processes, in congruence with Europe’s urge to promote growth and jobs, led to business oriented HCT/P legislation. In addition, we found indications that this specific example of EU safety and health legislation will actually adversely affect Member States’ health care systems.

## Methods

We analyzed the rationale and elaboration process of the recent EU HCT/P regulatory framework (EUCTDs and ATMP Regulation). Particular attention was paid to the following reports, which were provided by the EC and industry in support of policy making:

- Opinion No 11 of the European Group on Ethics in Science and New Technologies (EGE) to the EC on the ethical aspects of human tissue banking (EGE 1998). In December 1997 the EC set up the EGE to advise the Commission on ethical questions relating to sciences and new technologies. The EGE consists of up to 15 members, who serve in a personal capacity and are asked to advise the Commission independently from any outside influence (European Union 2009). The EGE cooperates with the Bureau of European Policy Advisors (BEPA), the Forum of National Ethics Councils (NEC Forum), the DGs concerned, representatives of the Institutions of the European Union, experts of the fields, parties representing different interests, including NGOs, patients and consumer organizations and industrial stakeholders.
- Written reports of the Working Groups established by the “Meeting on the Therapeutic Use of Human Organs and Tissues” organized by the Portuguese Presidency and the EC on 14–16 June 2000 in Porto (Loty et al. 2000). The aim of this meeting was to identify critical issues related to the therapeutic use of organs, tissues and cells of human origin, which either would need urgent follow up at Community level due to their implications, or for which different opinions among Member States pose difficulties for the development of common standards. The reports were drafted by groups of experts and were based on questionnaire-guided interviews of some hundred representatives from the public sector and industry.
- Proposal for a Directive of the European Parliament and of the Council on setting standards of quality and safety for the donation, procurement, testing, processing, storage, and distribution of human tissues and cells (European Union 2002a).
- EuropaBio’s (European Association for Bio-industries) proposals on DG Sanco’s proposed Directive regarding quality and safety of tissues and cells presented during a public hearing in the European Parliament on 29 January 2003. EuropaBio is said to represent more than 1,800 small and medium-sized enterprises (SMEs; EuropaBio 2002).
- Two evaluation studies carried out by the Directorate General Joint Research Centre’s Institute for Prospective Technological Studies (DG JRC-IPTS; Bock et al. 2003, 2005). DG JRC-IPTS was mandated by the “European strategy and action plan for life sciences and biotechnology,” which was developed to exploit the full potential of biotechnology and to strengthen the sector’s competitiveness while ensuring environmental and consumer safety and consistency with common values and ethical principles (European Union 2002b), to carry out biotechnology foresight with the objective of identifying newly emerging issues and possible proactive policy measures.
- Eucomed proposal for a Regulation of the European Parliament and of the Council on Advanced Therapies and amending Regulation (EC) No 726/2004 (Eucomed 2004). Eucomed represents the medical technology industry in Europe. Its mission is to make modern, innovative and reliable medical technology available to more people. Eucomed members include both national and pan-European trade and product associations as well as medical technology manufacturers. The industry they represent is said to employ more than 500,000 people.
- Eucomed position paper on the proposal for a Community regulatory framework on advanced therapies of 04 May 2005 (Eucomed 2005).
- ATMP Regulation impact assessment (IA) report (European Union 2005a). The IA process is one of the key tools put forward by the Commission to promote “Better Regulation for Growth and Jobs in the European Union” (European Union 2005b, c). It aims to assess economic, environmental and social impacts of EU policy. The IA report provides a detailed overview of the policy options envisaged by the EC with a view to establishing a harmonized regulatory framework for tissue engineered products, in the broader context of advanced therapies. It outlines the background to the proposal and presents an in-depth analysis of all legislative options available and possible impacts that may derive from them.
- Report of the stakeholder meeting organized by EuropaBio in Brussels on 9 November 2005 to discuss the proposed new Regulation on Advanced Therapies, prior to it being published by DG Enterprise (Geesink 2005). Some hundred participants from all over Europe attended this industry hearing, which followed a 2-day conference on commercializing tissue engineering and regenerative medicine. Major industrial players were represented, in addition to professionals from government, research and consultancy.
- Eucomed advanced therapy medicinal products backgrounder (Eucomed 2008).

We matched the prospective evaluations and perspectives forwarded by the above-mentioned reports against the retrospective and present states of the field, with an emphasis on Belgium. Incidentally, a Belgian company produces the first (and only) ATMP to have obtained both centralized European marketing authorization and national reimbursement (TiGenix 2011). The participation of industry in the policy making process (studies, consultations and IA) was also analyzed.

## Results and discussion

### The making of the EU HCT/P legislation

#### A regulatory survey

During a meeting convened under the Portuguese Presidency in Porto in June 2000, experts in the areas of organs, tissues and cells analyzed the regulatory situation in Europe (Table 1—2.1-8). A survey of existing regulation in EU Member States revealed several oppositions on ethical aspects, many similarities on safety aspects, but also a lack of regulation in many countries. The “Tissue Working Group” concluded that there is an urgent need for a single EU regulation on the quality, safety, traceability and vigilance of human cells and tissues (Loty et al. 2000). They also provided specific orientations for the development of such an initiative. A mother Directive should address general principles, while detailed annexes should address certain issues like quality and safety aspects. They also recognized the need for specific standards and inspections for each type of tissues or cells. Regulation of structures and activities could be performed at national or central European level (e.g. traditional tissues at national level, innovative tissues by central European level). The Organs Working Group agreed that legal initiatives should address the shortage of organs and tissues and that no new legislation should be enacted that limits the availability of living and cadaveric donors. Subsequently, experts and official representatives of the Member States arrived at a similar conclusion (EurActiv 2002). They supported the idea of developing an EC Directive setting high standards of safety and quality for the procurement, testing, processing, storage, and distribution of human tissues and cells in order to ensure a high level of human health protection in the EU.

#### A two-tier approach

From the beginning it was admitted that a number of new products that are based on a biotechnology process would profit from a different and specific regulation and legal basis. That’s why the HCT/P legislation was divided into two parts. One part—a Directive—would cover tissues and cells that are not “substantially manipulated” and are not part of a biotech process, so mainly for “traditional” transplants; the other part—a Regulation—would cover products and therapies that are subject to biotech processes that not only require specific regulation, but that also need a complete harmonization of requirements to facilitate their access to the market. The level of manipulation would thus determine if a graft is classified as traditional transplant or commercial product. It is thus not surprising that distinguishing between minimally and substantially manipulated proved to be contentious and problematic (Kent 2005). DG Enterprise decided to follow the FDA approach in what would be considered as substantial manipulations for hTEPs (Table 1—10.1).

#### Article 152 of the Amsterdam Treaty

The EU is based on the rule of law. This means that every EU action is founded on treaties, which have been approved voluntarily and democratically by all EU Member States. Article 152 (4)(a) of the Amsterdam Treaty authorizes the EC to install a regulatory framework for setting high standards of quality and safety of organs and substances of human origin (European Union 1997). This was as it were, the legal basis for the Commission’s interference in the HCT/P transplantation field (Table 1—2.1,3.1). In accordance with the principles of subsidiarity and proportionality, ECs actions in the public health sector should be undertaken only if their objective cannot be sufficiently achieved by the Member States and can therefore, by reason of their scale and effects, be better achieved by the EC. Community public health action shall, however, fully respect the responsibilities of the Member States for the organization and delivery of health services and medical care.

#### The EUCTD proposal

In 2002 DG Sanco (consumer health) drafted a Directive proposal (European Union 2002a) in line with Article 152 of the Amsterdam Treaty (Table 1—3.1) and taking into account the most recent progress made and agreements attained at international level, particularly within the World Health Organization (WHO) and the Council of Europe. In addition, there have been a number of consultations with competent technical experts and representatives of the Member States. According to the proposal, most of the organizations interested in the field were consulted, such as the European Association of Tissue Banks, the European Association of Musculoskeletal Transplantation, the European Eye Bank Association, the European Group for Bone Marrow Transplantation, the Donor Bone Marrow Association, Europdonor Foundation and the International Alliance of Patients’ Organizations. For the industry, Eucomed medical technology, the European Federation of Pharmaceutical Industry Associations and Baxter BioScience, a company offering GMP (Good Manufacturing Practice) biopharmaceutical manufacturing services, were invited to Stakeholders meetings. The measures set out in the proposed Directive incorporated requirements for the procurement, testing, processing, storage, and distribution of tissues and cells of human origin intended for application in the human body. In contrast to Regulations, Directives leave Member States with a certain amount of leeway, e.g. to introduce more stringent protective measures at national levels in conformity with the Treaty (Table 1—3.2). The Directive would apply to all constituents of the human body used for transplantation, except autologous cells used for medicinal products and cells and tissues used as autografts within the same surgical procedure (Table 1—3.4). The absence of profit by establishments involved in tissue and cell transplantation services was encouraged (Table 1—3.5). Because the EUCTDs would be enacted through common quality and safety standards and were thus merely seen as “technical matters” they evaded public debate (Hoeyer 2010). In addition, as it is only since 2005 that the current formal IA process became mandatory of all major EU policies, the IA of the Directive proposal was non-exhaustive and limited to the evaluation of its impact on business with special reference to SMEs. The main conclusion of the IA was that the requirements of this Directive could increase the cost for starting materials used by business and that no specific provision was envisaged for SMEs (Table 1—3.6-7).

#### Processing of the proposal

On 26 June 2002 the proposal was transmitted to the European Parliament (EP) and the Council. The proposal was submitted to the ordinary legislative procedure (ex “codecision”), which brings together Council, Parliament and Commission and has become the standard way of decision-making. This means that the directly elected EP has to approve EU legislation together with the Council (the governments of the 27 EU countries). The Commission, in turn, drafts and implements EU legislation. Debates during a 2003 EU parliamentary hearing on the proposal focused mainly on quality, safety and ethical concerns. Participants in the debate included representatives from industry (Eucomed and EuropaBio), scientists, commission officials, non-governmental organizations (NGOs), private blood banks and religious and bioethics organizations (Kent et al. 2006). EuropaBio put forth that companies’ expertise in development of innovative products warrants them to be accredited as tissue banks and that conflict (with the Treaty) would occur when Member States would not grant accreditation as tissue bank to industry (Table 1—4.1). Moreover, industry tissue banks should be able to move away from the traditional mode of tissue banking (Table 1—4.2-3). EuropaBio also called for the harmonization of the scope of the Directive for autologous and allogeneic cells used for industrially manufactured products for medical use (Table 1—4.4). The EP forwarded its opinion to the EC on 10 April 2003 (European Union 2003a). Most of EP’s proposed amendments suggested the strengthening of the Directive’s ethical provisions. Although recognizing their legitimacy, the Commission was unable to accept their inclusion in the proposal as ethical aspects fall outside the scope of Article 152 (European Union 2002c). On 30 May 2003 the Commission transmitted an amended proposal that took into account 35 of the 76 amendments. The Council subsequently endorsed the general approach taken in the amended proposal, adopting 15 of the 35 amendments (European Union 2003b). One of these amendments widened the scope of the Directive to autologous cells to be used for medicinal products. Some phrases in the amendments were corrected (e.g. “encouragement of the absence of profit by establishments involved into tissue and cell transplantation services” was changed into “Member States are urged to take steps to encourage a strong public and non-profit sector involvement into the provision of tissue and cell transplant services and the related research development”) to clarify that the goal is not to keep out the private sector. Some of the amendments that were not withheld addressed ethical issues such as voluntary and unpaid procurement (1), non-profit procurement (5), consent (3) or ethics in general (3) (European Union 2002c). One rejected amendment called for a code of conduct to protect human dignity and a ban on making the human body or its parts a source of financial gain, while several others basically proposed compliance with fundamental ethical principles next to compliance with quality and safety standards. The common position adopted on 22 July 2003 followed the same direction on the EP’s amendments as that of the Commission—accepting the majority of those related to technical aspects and, given the perceived absence of a legal basis, rejecting those dealing with ethics. On 31 March 2004 the EP and the Council adopted the parent Directive 2004/23/EC of the EUCTDs (European Union 2004). In conclusion, the principle of subsidiarity was adopted as a way of evading ethical issues (e.g. with regard to the use of human tissue to make profit) and enabling national interests to be accommodated. At first sight, industry’s lobby for changes to the Directive that would allow them to procure, store and process tissue and to be accredited as a tissue establishment was fairly successful. However, as we shall see further in this paper, in some Members States it is still impossible for industry to carry out fully fledged tissue banking activities. As provided in the initial two-tier plan, the EUCTDs would thus *pave the way* for the high quality and safe application of human tissues and cells in therapies which use the method of tissue engineering (EurActiv 2004a).

#### The Lisbon Strategy and the open method of coordination

In March 2000, at the European Council in Lisbon, the EU set itself a new strategic goal for the next decade: to become the most competitive and dynamic knowledge based economy in the world capable of sustainable economic growth with more and better jobs and greater social cohesion (European Union 2000). Under this so-called “Lisbon Strategy,” the Commission recalled the economic, social and environmental potential of life sciences and biotechnology—which was said to have entered a stage of exponential growth—and, in consequence, the strategic and long-term importance for Europe of mastering these sciences and technologies and their applications (European Union 2001b). Europe could not afford to miss the opportunity that these new sciences and technologies were to offer. In 2004, the Lisbon strategy was reviewed and it was concluded that even if some progress was made, most of the goals were not achieved (EurActiv 2004b). The EC subsequently issued a proposal to refocus the Lisbon Strategy on actions that promote growth and jobs (Baroso 2005). To overcome the implementation gap identified during the review, a relatively new and intergovernmental means of governance was inaugurated: the open method of coordination (OMC). It is a decentralized approach through which agreed policies are largely implemented by the Member States and supervised by the Council of the EU. Formally, the EC has primarily a monitoring role, but in practice it helps to set the policy agenda and persuades reluctant Member States to implement agreed policies (Flear 2009). Today, OMC is the preferred method for EU action in sensitive policy areas at the core of national sovereignty, including health care. The objectives of the OMC serve as guideline for national policy. They valorize market rationality and begin the reframing exercise by defining what is to be achieved (Flear 2009). Member States are then assessed, and placed in a hierarchy of progress, through the use of the objectives established in the OMC. In this, the Commission uses its ability to muster expert views and indicators and its position as a hub of the OMC process. Finally, the EC persuades the “bad pupils” to implement the agreed policies, even if they technically belong to the competence of the Member States (e.g. the OMC on health care).

#### Mind the (premeditated) gap

In the late nineties the emergence of human tissue-engineered technologies was accompanied by debate about the governance of this field. There was a widely, though not unanimously, perceived need for a new harmonized regulation (Faulkner et al. 2006). Industry suggested that in the absence of such a pan European regulation for cell- and tissue-based products EU patients would be denied the potential benefits of this regenerative medicine. Industry is generally in favor of harmonized legislation as it creates predictability, helps to make informed investment choices (Table 1—4.5) and reduces the cost of having to meet different quality, safety, efficacy and marketing requirements (Kent et al. 2006). Of course, for the EC to intervene, this regulatory gap needed to be documented and confirmed by independent experts. In 2003, a DG JRC-IPTS study (Bock et al. 2003) confirmed that because hTEPs differ in many ways from medical devices and pharmaceuticals, they lay outside of any EU legislative framework (Table 1—5.5). Indeed, they were explicitly excluded from the scope of medical devices Directive (European Union 1993), and the medicinal products Directive (European Union 2001a), which regulates gene therapy medicinal products (GTMPs) and somatic cell therapy medicinal products (sCTMPs), but not hTEPs. In turn, the EUCTDs did not lay down rules for the marketing of HCT/Ps (Table 1—3.3) nor did they mention *efficacy* criteria (Table 1—9.10). This is mainly because they were based on Article 152 of the EC Treaty, which aimed at establishing a high level of human health protection while respecting the responsibilities of the Member States for the organization and delivery of health services and medical care, but did not pursue an “internal market” objective (EurActiv 2002). Moreover, from the start, the EC had decided that products and therapies that are subject to biotech processes and need a complete harmonization of requirements to facilitate their access to the market would be covered by a dedicated Regulation. The exclusion of hTEPs from the EUCTDs—the regulatory gap—was premeditated.

#### Public consultations

DG Enterprise conducted two public consultation rounds (in 2002 and 2004), which revealed a disagreement about whether dedicated legislation is needed for hTEPs or whether revisions to the existing medicinal products or medical devices Directives would be more appropriate (Kent et al. 2006). Government/institutional officials favored using the existing framework and EMA felt that the existing framework for medicinal products should be used, supplemented as necessary by the framework for medical devices. In contrast, industry supported a new legal framework. Industry highlighted the distinctiveness of hTEPs, their diversity and a view that existing medicinal product regulation is too restrictive, costly and that lengthy product approval times would limit the ability of industry to bring these newer products to the market (Kent et al. 2006). In the end, the consensus was that public consultations had resulted in the expression of a clear need for a *specific* Community framework to cover hTEPs, taking into account a tiered approach depending on the level of risk.

#### The hTEP regulation proposal

Following the public consultations, DG Enterprise (not DG SANCO) prepared an hTEP Regulation proposal (European Union 2005d) intended to bridge the regulatory gap identified by the DG JRC-IPTS studies (Bock et al. 2003, 2005). The draft Regulation had as main objectives to secure a high level of health protection, to harmonize and facilitate internal market access and finally to foster competitiveness. DG Enterprise was required to address Article 95 of the Amsterdam Treaty (EurActiv 2002) related to the free movement of products across the EU. The proposal incorporated requirements such as production according to GMP and compliance with marketing authorization requirements and post-marketing pharmacovigilance rules. The biotech industry, which had been calling for a Regulation such as this for years, welcomed the proposal (Pincock 2005). One particularly positive note perceived by industry was the Commission’s proposal to have the regulatory process centralized through EMA. In addition, the Commission’s plan also involved strengthening requirements for risk management and traceability of gene, cell and tissue-based therapies, and offered special incentives for SMEs working in the field (Pincock 2005).

#### The hTEP draft regulation IA

In 2005, a DG JRC-IPTS study (Bock et al. 2005) set out to identify and assess the economic, social and environmental impacts of several regulatory options presented in the hTEP draft regulation, as an input to a formal IA (European Union 2005a). According to the IPTS study and IA reports, the lack of a tailor-made and uniform EU legislation would lead to divergent national Member State approaches as to the legal classification and authorization of hTEPs, which impaired the free movement of these products, deprive patients’ access to innovative therapies using hTEPs, acted as barriers to guaranteeing a high level of public health protection across the EU and ultimately hampered the development of a strong tissue engineering sector in the EU and affected the EU competitiveness in this key biotechnology area (Table 1—9.2). The overall conclusion of the IA was that the proposed regulation would be of significant benefit for all actors in the field by providing legal clarity and certainty, harmonizing quality and efficacy standards for the placing on the Community market of hTEPs, improving the competitiveness of the concerned economic operators and increasing the confidence of patients and healthcare practitioners. The draft regulation was released for an additional public consultation in May 2005. Meanwhile, the EC decided to expand the hTEP regulatory text to also cover other ATMPs, like GTMPs and sCTMPs.

#### Processing of the proposal

Because the Commission conceived the ATMP Regulation as a matter of science, technology, regulation and the operation of the internal market, the proposal appeared to be an ideal candidate for an “early agreement” in the codecision procedure between the EP and Council, which had become the standard fast track—but less transparent—EU lawmaking procedure (Judge and Earnshaw 2011). The dossier was indeed concluded at first reading, but not without generating significant political conflict and intense controversy over ethics (as was the case for the EUCTDs) during its passage through the EP. Ethical issues that were extensively discussed and lobbied included perceived breaches of the principles of the non-commercialization of the human body, the integrity of the person and the inviolability of human dignity. In the end, ethical amendments were deemed controversial and were dropped (Judge and Earnshaw 2011). On 30 October 2007 the Council formally adopted the Regulation, which was signed by the EP and Council presidents on 13 November 2007 (European Union 2007). On 30 December 2008, the ATMP Regulation entered into force. Tissue engineered products that were legally on the Community market in accordance with national or Community legislation on 30 December 2008 should comply with this Regulation no later than 30 December 2012.

### Industry’s influence on the regulation and policy processes

For the elaboration of EU legislation policymakers rely on key decision-making tools such as knowledge and expertise, consultations of stakeholders and an IA process.

#### Knowledge and expertise

Policymakers often rely on consultancy firms, which are simultaneously working for commercial companies, for their supply of knowledge and expertise. Both DG JRC-IPTS hTEP studies were carried out in collaboration with the European Science and Technology Observatory and in particular with the Fraunhofer Institute for Systems and Innovation Research, part of the Fraunhofer Society, the largest organization for applied research in Europe. According to their 2011 annual report (Fraunhofer-Gesellschaft 2012), over 70 % of their contract research revenue is derived from contracts with industry and public sector research projects. The remaining 30 % comes from the federal and Länder governments, among other things to finance precompetitive research projects of direct benefit to both industry and society.

#### Consultation of stakeholders

In preparing EU legislation, policymakers are required to consult all potentially affected stakeholders. But, most public institutions seem to have underestimated the scope and thus also the impact of EU legislation. In addition, they had limited resources and were unaware or unable to fully participate in the consultation processes. As a result, companies are often overrepresented in these consultations (Smith et al. 2010a). Consultations in view of the ATMP regulation included workshops and round table meetings, stakeholders’ interviews by DG JRC-IPTS and public consultations. While 117 tissue engineering companies from 14 countries (70 % of companies having products on the markets), EMA, and all NCAs were involved in the consultation process, for hospitals and tissue banks only a limited survey was carried out. Only 21 questionnaire-guided interviews (30–60 min duration) were performed with relevant experts and representatives from hospitals and tissue banks in only 3 countries (Germany, the UK and France; Bock et al. 2003, 2005). Competent authorities for medicines were also consulted. They are perceived to be independent, i.e. not to represent any government, organization or sector and therefor there is a tendency to particularly value their advice. Yet, they are increasingly funded by the pharmaceutical industry. EMA’s budget is financed both from the EU’s annual budget, and to a greater (and increasing) extent (80.1 % in 2011) from fees paid by pharmaceutical companies (EMA 2012). Recently, EMA was accused of acting to promote the interest of the pharmaceutical industry. In 2011, the EP officially declared: “it is unacceptable that the Agency does not apply the relevant rules effectively, resulting in the fact that there is no guarantee that the evaluation of human medicines is performed by independent experts” (European Union 2010b, 2011). The 2010 annual report of the Belgian NCA for medicines shows that more than half of its income comes from taxes collected on the basis of the number of packages of medicines and raw materials sold or on the turnover generated from medical devices (FAMHP 2011).

#### The IA process

The current IA process was designed to allow policymakers to assess the likely effects of potential options, in advance of their implementation, on the basis of careful analysis of the potential economic, social and environmental impacts of new legislation. Health impacts are subsumed in social IA. The current form of IA has been criticized for favoring economic impacts over environmental or social (and particularly health) impacts. Recently, it was demonstrated that lobbying efforts by an alliance of corporate actors have helped promote and embed a system of IA in the EU that is business orientated, for example encouraging policymakers to consult business (Smith et al. 2010b). This increases the likelihood that the EU produces policies that advance the interests of major corporations, including those that produce products damaging health, rather than in the interest of its citizens (Smith et al. 2010b). In current IA it is easier to predict and prioritize positive economic and business-related impacts over less tangible, long-term negative impacts relating to health (Smith et al. 2010b). For example, the IA process uses monetized values to predict impacts, which can be problematic because there is no agreed way to value some of the most fundamental health impacts, such as lives saved.

#### Industry’s lobby

The participation of industry in key decision-making tools was thus overwhelming. But, the lobbying exercised by industry is not illegal. Moreover, trade federations often regard their participation as “offering help for the elaboration of guidance documents” (Table 1—8.1). For the EC “lobbying” means all activities carried out with the objective of influencing the policy formulation and decision making processes of the European institutions. Concerns have however been voiced by the media, academia and interest representatives about lobbying practices which are considered to go beyond legitimate representation of interests. This applies amongst others to some improper or misleading lobbying methods (European Union 2006c). The examples that are often quoted in this context are the provision of distorted information to the EU institutions about the possible economic, social or environmental impact of draft legislative proposals (European Union 2006c).

### The new “EU-style” HCT/P transplantation field

**Fig. 1:**
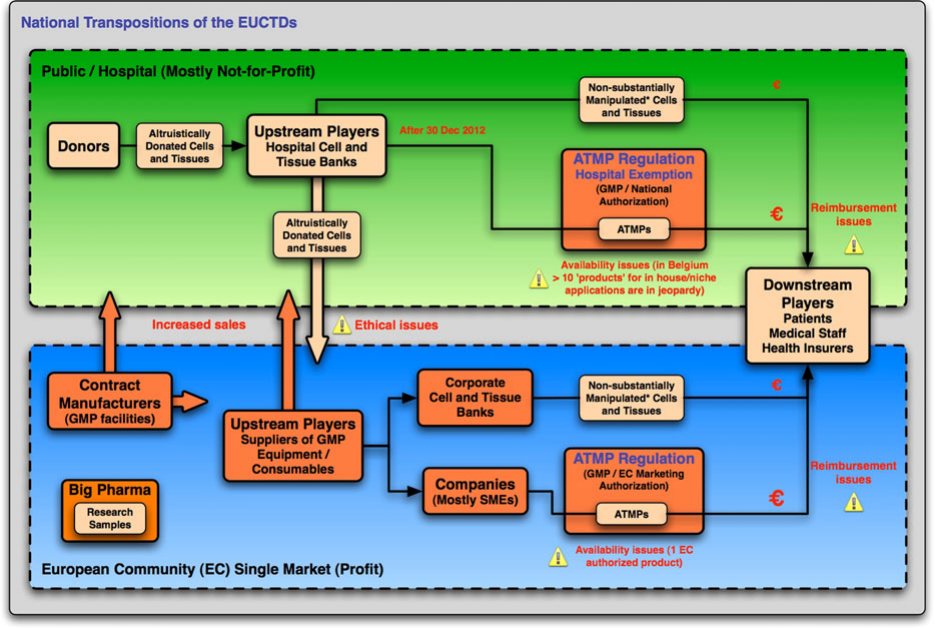
The HCT/P transplantation field as devised by the EU

With the new HCT/P regulatory regime (EUCTDs and ATMP Regulation), the EC finally opened the door to the commercialization of human cells and tissues. The commercial sector was given a central role in a single HCT/P market (Fig. 1). Hospitals, or “upstream players” as they are called in official EC documents, are reduced to providers of starting materials (human cells and tissues) to the industry. Patients are “downstream players,” research subjects or consumers (eventually self-paying, if there is no other way) of the products provided by the industry (which are not necessary those asked for by clinicians). Academia should research and develop the innovative HCT/Ps commissioned by industry and funded by EU agencies, which increasingly prioritize health research in support of industry. The ATMP IA report explains that hospitals and tissue banks should collaborate with industry, or better, become providers of human cells and tissues to the emerging tissue engineering industry (Table 1—9.8). These providers only have to comply with the provisions laid down in the EUCTDs. Strangely, this means that the production of the “raw materials” or “starting materials” used to produce ATMPs does not need to be compliant with established GMP. This scenario benefits to tissue(-engineering) companies, which today are confronted with a limited supply of starting materials (donor cells and tissues). For example, according to CNN Money, the product Alloderm™ (a skin substitute derived from human cadaveric skin, which earned LifeCell the 16th place on FORTUNE’s 100 Fastest-Growing Companies list in 2004) has only one hitch: raw material (human donor skin) supply constraints (Birger 2006). The not-for-profit image of public tissue banks and laboratories does not negatively influence the willingness to donate cells and tissues and makes them the ideal suppliers for private companies, which are generally distrusted by the public. Altruistic cell and tissue institutions are at risk of being reduced to facades behind which controversial commercial HCT/P activities can be hidden from the public.

### Ethical issues

#### Tradable goods

The EC considered HCT/Ps to be tradable goods and as such they were to be governed by the European Treaties and Directives. Normally, health related products are subject to the authority of the individual Member States due to the subsidiarity for health care matters (Article 152 (4)(c) of the Amsterdam Treaty) (European Union 1997; Trommelmans et al. 2007a). But, Article 152 (4)(a) of the Amsterdam Treaty authorized the EC to install a regulatory framework for setting high standards of quality and safety of organs and substances of human origin (European Union 1997). As could be expected, various stakeholders presented a wide variety of philosophical, social, religious and economic viewpoints on relevant ethical issues and in particular on the prohibition of commercialization and commodification of human bodily material, which lead to fierce ethical debates (particularly in the EP) throughout the elaboration process of the regulatory framework. For some stakeholders tissues originating from an altruistic (free) donation should only be handled by non-profit-making cell and tissue banks and laboratories, while others argued that the processing of tissues (into hTEPs) involves costs, which justify their commercialization, which provides an incentive for industry to invest in tissue engineering. When consulted on the hTEP draft regulation, the EGE reworked an earlier opinion (1998) in which it acknowledged that the issue of commercialization of human tissues, which have been processed and prepared for therapeutic purposes, might be controversial (Table 1—1.1-3), but concluded: “it is difficult to exclude tissue banking activities by commercial organizations, particularly where human tissues are used as a basis for ‘engineered’ products requiring the use of sophisticated medical techniques” (Table 1—1.5). EGE’s opinions had no legal power as such, but they strongly influenced the ongoing debates. Already in 2000 they served as a reference during the meeting on the therapeutic use of human organs and tissues (Table 1—2.2) and they were part of the IA that accompanied the ATMP draft Regulation (Table 1—9.1).

We identified two main ethical principles that are applicable to the current HCT/P transplantation field: the basic principle of “respect for human dignity” and the principle that “human bodily material should not be considered as a commercial product or a commodity,” the latter deriving its moral force from the former. The key question is: will the processing (engineering) of human cells and tissues lead to a *product* that is no longer subject to these ethical principles? Can processing alter the moral status of human bodily material? One could consider HCT/Ps to be “dual products,” consisting of human bodily material and an added value in the form of a technological process. Both parts clearly have a different moral status, which leads to an ethical dilemma; the human bodily material is not a tradable good, while the added technological process (know-how) clearly is. The problem is that one cannot be sold without the other. A possible way out of this dilemma would be to use the “doctrine of double effect (Cavanaugh 2006):” if an action has foreseen harmful effects practically inseparable from the good effect, it is justifiable if the following are true:

- the nature of the act is itself good, or at least morally neutral;
- the agent intends the good effect and not the bad either as a means to the good or as an end itself;
- the good effect outweighs the bad effect in circumstances sufficiently grave to justify causing the bad effect and the agent exercises due diligence to minimize the harm.

Translated to the HCT/P field, this could imply that the commercialization of human bodily material (foreseen harmful effect) could be justified when tissue establishments act in good faith and produce HCT/Ps for use in meaningful (e.g. life-saving) therapies (good effect in grave circumstances). The good faith of tissue establishments could be reflected in a HCT/P cost price that only relates to the added technological process, and this in a reasonable manner. This avenue should be examined in greater depth, but this is beyond the scope of this paper.

#### Ethical issues evaded

It was suggested that, from the start, ethical issues must be considered as an integral part of the legislation (Trommelmans et al. 2007b). But, according to the official discourses, some ethical issues, such as the eventual access of commercial companies to human bodily material, were strongly affected by national culture, and therefore it was deemed too hard to negotiate a proposal that dealt with ethical issues and that would have been backed by a Council composed of 27 National Health Ministers. According to the EC, issues raised by biotechnology should be addressed at the appropriate level in accordance with the subsidiarity principle (European Union 2001b). The EC has a clear responsibility in some areas concerning trade and internal market implications, while the responsibility on setting the ethical principles lies with the Member States (European Union 2001b). Technically, ethical issues were deemed legitimate, but out of the scope of Article 152 (4)(a): i.e. the quality and safety of organs and substances of human origin. One could, however, argue that ethical issues such as paid or unpaid donation, the type and extent of donor consent and whether or not to commercialize human bodily material can definitely impact the quality and safety of HCT/Ps. The principle of subsidiarity was thus adopted as a way of evading ethical issues. This “cultural ethical relativism” (each culture should use its own standards to judge all actions and institutions) is not immediately obvious when it comes to the field of healthcare, because one may assume that health is a universal ethical good. In addition, similar ethical issues in the organ transplantation field, where industry plays a less pronounced role, are dealt with on a global level (Steering Committee of the Istanbul Summit 2008). There is a need for a *global* and binding ethical framework for human cell and tissue product transplantation that prohibits financial gain on the human body and its parts (Pirnay et al. 2010). For a start, the EU should adopt a clear ethical position overcoming commercialization issues.

#### Responsibility towards the donor

The HCT/P transplantation field put forward by the EC (Fig. 1) disregards the ethical issues related with the supply of altruistically donated cell and tissues to commercial companies. In certain cases, this transfer might be in conflict with the public cell and tissue banks’ mission statements and with their responsibility towards the donor or donor family to process the cells and tissues in a manner consistent with the intent of the donor, i.e. into products that fulfill medical needs, crucial research or medical education. This is all the more true in Member States that apply the “opting-out” system (presumed consent) for organ and tissue donation. Public cell and tissue banks that transfer donor material to private companies should thus at least inform the donor family of the foreseeable commercial exploitation and/or secondary use (e.g. in cosmetic or vanity procedures) of the donated cells and tissues (Office of Inspector General 2001). According to the ATMP IA report, few stakeholders requested that the donor be informed of the usage made of the tissue, which they provide as source material (Table 1—9.13). What if a donor (or his family) *voluntarily and knowingly* donates bodily material (for free or against payment) to a tissue broker or establishment that will sell it as starting material for the production of HCT/Ps? Although this seems to be in line with the principle of autonomy, which recognizes the right of individuals to self-determination, it goes against the basic principles of “respect for human dignity” and “non-commercialization of human bodily material.” Indeed, it could be argued that individual autonomy presupposes a so-called liberty-right to decide freely on the use of one’s own human material. In our view, however, this is not so: autonomy is not just about an unlimited freedom to decide and act as one feels like. Being an autonomous person is also about being a morally responsible person. In our case, such a morally responsible individual would readily recognize that, informed by the two basic principles, human cells and tissues (one’s own or somebody else’s) should not be degraded to tradable goods. In addition, interventions that infringe on individual autonomy may be necessary to secure public health (i.e. the health of the entire population). For example, the decision of individuals to donate skin to companies that transform human skin into products for use in meaningless therapy or even in vanity procedures (e.g. lip enhancements) could be overruled on the basis that this practice could lead to a (local) shortage of donor skin for use in life-saving burn wound surgery and could thus impact public health. More importantly, in people’s minds public donation is all about life saving gifts, and donation could stop straight away if the public felt abused.

The bottom line for all ethical guidance in the HCT/P transplantation field should however remain the commitment of regulatory agencies and of HCT/P producers and researchers to fundamental values: equitable access of patients to safe and efficacious grafts, respect for the autonomy and the rights of cell and tissue donors, and respect for the dignity of all involved in HCT/P transplantation (Trommelmans et al. 2007a).

### Technical requirements

#### GMP

In 2006, the HCT/P transplantation field—a formerly non-industrial environment—was first confronted with pharmaceutical industry requirements. The EUCTDs, and more specifically Technical Directive 2006/86/EC (European Union 2006b), introduced GMP requirements, which were limited to the air quality of the processing facility. A formal GMP (cleanroom) facility was not required for the processing of HCT/Ps. Since 30 December 2012, however, the ATMP Regulation implies GMP compliance in a general sense to a subgroup of HCT/Ps. Yet, GMP knowledge is sparse in academia and hospitals; it has traditionally resided in pharmacy (Hildebrandt and Sethe 2012). Some academics pretend that GMP originates from industry scandals due to excessive profit-maximizing activities. The incentive to cut corners to maximize profits, and thus negatively impact public health, is expected to be higher in the profit sector. Conversely, some industry representatives argue that academia have a history of side stepping quality and safety rules. The truth is that compliance with certain quality and safety requirements is imperative to protect patients, industry and academia alike from abuses (e.g. charlatans offering unproven stem cell therapies on the internet; Hildebrandt 2012). The ATMP IA report (Geesink 2005) stresses that guidelines on the application of GMP and good clinical practice (GCP) for ATMPs should be drafted in close consultation with all interested parties and in particular with industry (Table 1—9.9) and Eucomed subsequently underlined that medicinal product GMPs are not directly applicable to hTEPs and will need to be redesigned (Table 1—10.9).

#### The precautionary principle

The key question here is whether the recently implemented (30 December 2012) higher level manufacturing requirements for ATMPs will really improve their quality and safety? To begin with, we are not aware of a thorough scientific evaluation of the quality and safety aspects of HCT/Ps (including the ATMP subclass) since their mandatory compliance with the technical components of the EUCTDs on 1 September 2007. We are not aware of any report of safety issues associated with ATMPs that up till now only needed to be compliant with the EUCTDs. Yet, it is assumed that supplementary manufacturing requirements are imperative for public health. In earlier times, quality and safety requirements used to be evidence based, scientifically and clinically justified. It is odd that in an age of “evidence based medicine,” regulators increasingly rely on the precautionary principle—i.e. the prevention of harm to human health by removing the requirement for scientific proof of risk in advance of legislative intervention, thus evading liability (umbrella policy) and shifting the burden of proof to the researchers and manufacturers. This results in overzealous technical requirements, which are not bad per se, if it weren’t for the disproportionate costs. In 2005, EuropaBio’s Secretary General rightfully said that three words were very important today: cost, cost and cost. He warned to be very careful not to kill the SME or the product with demands that go over the capacity (Table 1—10.15). There is a point at which legislation can actually compromise patient care and safety, by disabling valuable established therapies or delaying the development of new technologies. According to Kirkland (2010), we should try to balance the risk avoidance principles with the broader risks to the community that can result from overzealous or inappropriate application of regulatory standards. To quote Alastair Kent, Director of Genetic Alliance UK: “perfect is the enemy of the good” and “if we eliminate risk, there will be no progress.” A considerable risk is sometimes worth taking. For example, a parachute that only opens in 90 % of cases is not acceptable in normal circumstances. But, what if the pilot died and the airplane is 100 % sure to crash? Patients should be more involved in the decision making process as Regulations must follow the biology of patients (Kent 2012). According to Richard Woodfield from the United Kingdom (UK) Medicines and Healthcare Product Regulatory Agency (MHRA), technical requirements need to be risk based and fully proportionate, to reflect the characteristics of the individual product (Table 1—10.3). For some HCT/Ps, risk-based approaches for bioburden control in non-sterile products could be acceptable (Migliaccio 2012). Donor skin (products) for use in burn wound patients, for example, do not need to be sterile and do not need to be processed in a clean room facility (Pirnay et al. 2012b). Recently, DG Sanco forwarded a revised version of Annex 2 of the GMP Guide to accommodate for several new product types, including ATMPs (European Union 2012). Interestingly, in some cases marketing authorization or clinical trial authorization provides for an allowable type and level of bioburden instead of sterility.

#### Enforcement

Higher level technical requirements such as GMP compliance do not guarantee—nor are they a prerequisite for—quality, safety and efficacy of a product. Of course, Directives and Regulations are in themselves insufficient to prevent patients from unsafe medical products or devices. The PIP breast implant scandal (Donawa and Gray 2012) is a recent illustration of this. A French company decided to downright ignore the applicable quality and safety standards by making breast implants from cheaper industrial-grade silicone normally used for electronics, mattresses or the agriculture industry (Chrisafis 2011). Yet, the company was in possession of a certificate of conformity with European standards (including compliance with GMP for medical devices) and hundreds of thousands of implants were sold and implanted on three continents. There is still a lack of inspection and effectiveness measures. We feel that common sense and enforcement of the EUCTD requirements through adequate inspections are more important for patient safety than imposing GMP compliance to the HCT/P transplantation field. Within the European Standards and Training in the Inspection of Tissue Establishments (EUSTITE) project, inspector-training courses were started in 2008. A unitized valuation during the inspections will be an important step to get the aim of similar quality and safety of human tissues and cells that are applied to patients for therapeutic purposes in the Member States (European Union 2010c).

### Private versus public tissue establishments

#### Competition

Since the introduction of the Lisbon Strategy, competition between health care providers is seen as a means to reduce costs and increase quality. According to the second DG JRC-IPTS study report, the current trend of concentration of tissue establishments and HCT/Ps due to adaptation to national and European standards (i.e. the EUCTDs) would continue. In the short term, manufacturers’ need to adapt to these more stringent quality and safety standards and requirements for marketing authorization would tie up resources. As well as forcing companies to concentrate on fewer products, this could tip the scales in favor of larger firms better able to target pan-European markets (Table 1—6.2). This prediction was repeated in the ATMP IA report. Of course, this scenario might also apply to hospitals and tissue banks active in tissue engineering. Moreover, according to the IPTS study and IA reports, hospitals and tissue banks could be regarded as competitors to tissue engineering companies, even if they only made their products available to patients in their country and occasionally beyond national borders (Table 1—9.3). According to the IA report, this potential competition would be open due to the often public, non-profit character of hospital tissue banks. Hospitals and tissue banks would have less marketing costs and would not bring in profit margins, whereas tissue-engineering companies could exploit economies of scale (Table 1—9.4). Further on, the IA minimalizes the impact of this competition by stating that it is expected to remain limited in the short to medium term (Table 1—9.5). In an animated discussion during the European Association of Tissue Banks Congress’ ATMP/HE Workshop (Vienna, 22 November 2012) EU policy makers and competent authorities asked the public cell and tissue banking community to empathize with tissue companies, which are asked to comply with expensive and stringent regulation while hospitals can use the HE loophole to produce cheaper “generic” versions of their products. But, isn’t this putting the cart before the horse? One could as easily argue that ATMPs were and still are mainly produced by public cell and tissue banks, which are now confronted with pharmaceutical requirements asked for by industry in order to appropriate the human cell and tissue transplantation field for themselves.

#### A level playing field

Initially, the ATMP Regulation proposal stated that the Regulation should not apply to any ATMP that is prepared by a qualified and licensed professional, such as a pharmacist, physician, or trained and certified biologist, on an exceptional basis, in order to comply with a medical prescription for an individual patient; the product must be prepared in full at the site of treatment of the patient, and without using standardised or patented processes. Eucomed lobbied to remove this exemption (Table 1—7.1). Industry insisted on a level HCT/P playing field, out of concern for public health (Table 1—7.1, 8.5, 10.2, 11.1). The ATMP IA report reveals that industry stakeholders considered the initially proposed definition of “placing on the market” as improper because it did not cover products manufactured and used in the same facility (in-house use, for instance in hospitals; Table 1—9.6). A large majority of stakeholders, including the European Parliament’s rapporteur for the EUCTDs (Table 1—10.7), were of the opinion that hospitals, tissue banks and other local actors should be subject to *similar* rules as enterprises. This is not surprising, considering that most consulted experts were indeed representing, or had affinity with industry. Stakeholders from the “healthcare professionals” and “research” category in turn argued that the exclusion was too narrow, that the concept of “industrial manufacturing process” may be too vague and that hospitals and university/research environments should not be imposed unnecessary regulatory overburdens such as marketing authorization requirements (Table 1—9.7). In the end, it was decided that all ATMPs, including those manufactured and used on single patients in a hospital, would fall within the scope of the ATMP Regulation.

### The current state of the HCT/P transplantation field

#### Gold plating

The term “gold plating” refers to the practice of national bodies exceeding the terms of EC directives when implementing them into national law. This is what happened with the EUCTDs. The official aim of these Directives was to set out harmonized quality and safety standards for dealing with human tissues and cells. The underlying aim was to provide access to safe cells and tissues for the emerging HCT/P industry—“access to primary material should be guaranteed and that manufacturers are allowed to settle themselves as tissue establishment” (Table 1—4.1). As expected, some Member States—mainly for ethical reasons—did not want to give that kind of direct access to industry. The principle of subsidiarity allowed them to add extra measures on top of the EUCTD requirements to prevent industry’s direct access to human cells and tissues (Table 1—3.2; Kent 2005). In Belgium, for example, only tissue banks exploited by a hospital can obtain direct access to human cells and tissues for allogeneic use. So, the de facto situation of this development is that companies targeting the EU market are confronted with potentially different systems for access to primary material (Table 1—10.11). This means that industry’s access to primary material (the underlying goal of the EUCTDs) is not guaranteed (Table 1—10.8, 11). According to the industry, this situation will lead to patients being denied promising treatments.

#### The actual players

According to the 2003 DG JRC-IPTS study report, there were only limited data available on the scope and extent of the tissue engineering activities of public tissue banks and laboratories (Table 1—5.4). Instead of actually producing the missing data, the research center chose to assume that hospitals carried out research or produced fairly simple, autologous hTEPs for in-house treatments and considered tissue engineering as a future strategic option, but did not yet produce any hTEPs (Table 1—5.4, 6.1). The commercial tissue-engineering sector in Europe was said to be characterized by small research-based technology-intensive biotechnology companies (Table 1—5.3). According to Eucomed, SMEs did indeed represent the large majority of manufacturers of hTEPs, but also big corporations would invest in this promising branch of medical technology (Table 1—8.3). Today, however, it is clear that with regard to ATMPs, the pharmaceutical industry has only limited interest in playing its “usual” role of financing development and acting as a sponsor in clinical trials (Hildebrandt and Sethe 2012). Several reasons for this were suggested, including intellectual property and reimbursement issues and the fact that ATMPs are more closely related to transplantation, an area that does not interface much with established industrial R&D (Hildebrandt and Sethe 2012). In contrast, the pharmaceutical industry is very much interested in obtaining valuable research cells and tissues (Barnes 2006). For example, human instead of animal tissue can be used in earlier stages of new drug testing to more accurately predict the safety of new treatments. Traditionally it has been hard for drug firms to get hold of human tissue due to ethical issues and the challenges of patient and family consent. As a result, human tissue is said to be worth more than diamonds, being valued at $500/g (Barnes 2006).

Today, it is clear that the actual developers of ATMPs are different from those of conventional medicinal products, with a very high proportion of ATMPs developed by academia/hospitals and SMEs and an almost complete absence of “big pharma” (No authors listed 2011). In contrast to the assumptions presented in the IPTS studies, public actors did certainly provide the majority of grafts that today have become ATMPs (Hildebrandt and Sethe 2012). In Belgium, as in most Member States, accredited cell and tissue banks adhered to national human cell and tissue legislations and quality standards long before the introduction of the EUCTDs. These national regulatory frameworks enabled the provision of acceptable amounts of affordable, safe and ethically sound transplants, including numerous grafts that today are considered to be ATMPs. On its website, the Belgian NCA for medicines maintains a list with approved Belgian establishments of human bodily material. According to this list (October 2012 update; FAMHP 2012a), 13 establishments were approved for “advanced therapy” (with reference to the ATMP regulation 1394/2007/EC) under the EUCTDs (Table 2). The majority (n = 10) of them are hospital tissue banks or laboratories, while only three are private companies. These 10 public establishments provided 22 grafts approved for use in advanced therapies (Table 2). Already in the nineties, years before both IPTS studies, 3 Belgian public tissue establishments were accredited by the Belgian NCA (upon inspection) to produce and distribute “hidden” ATMPs for clinical use (Table 2). We had to wait until 2009 for the first private company to get an ATMP on the market. Recently, the Belgian NCA summoned the accredited Belgian establishments of human bodily material to submit a letter of intent to declare and specify activities that could qualify as ATMP. A few days before the submission deadline (31 March 2012), 21 potential ATMP activities were declared (Mush 2012). A recent survey revealed that in Europe, 80 % of ATMPs are under development in academia (Hildebrandt 2012). Actually, SMEs and particularly public cell and tissue banks are essential for ATMP development and production because they are the only operators that will target grafts on niche markets (e.g. severe burns), which are not or less attractive for large players. Yet, their central role as drivers for the development and manufacture of ATMPs has been overlooked (Hildebrandt and Sethe 2012).

**Table 2:** Belgian establishments of human body material accredited (as published in Ministerial Decrees) for “advanced therapy” (with reference to the ATMP regulation 1394/2007/EC) under the EUCTDs, until 30 December 2012 (FAMHP 2012a)

Name	ATMP	Accredited by the Belgian Ministry of Health since
Public		
Liège University Hospital	Dendritic cells	4 December 2007
	Mesenchymal stem cells	4 December 2007
	Pre-osteoblastic cells	30 December 2008
South Luxemburg Hospital	Proliferative tissue	11 July 2011
Saint-Luc University Hospital	Hepatocytes	13 February 2006
	Hepatic stem cells	2 June 2008
	Islets of Langerhans	30 December 2008
	Adipose stem cells	25 August 2009
Institute Jules Bordet	Dendritic cells	30 December 2008
	Mesenchymal cells	30 December 2008
	Lymphocytes	1 December 2009
Antwerp University Hospital	Dendritic cells	30 December 2008
	Mesenchymal cells	30 December 2008
	Epithelial cells	30 December 2008
Brussels University Hospital	Beta cells	18 December 1997
Vrije Universiteit Brussel	Dendritic cells	30 December 2008
Ghent University Hospital	Dendritic cells	21 September 2010
	Keratinocytes	18 December 1997
Leuven University Hospital	Mesenchymal stem cells	1 September 2011
	Dendritic cells	5 August 2010
	Keratinocytes	17 February 2000
Queen Astrid Military Hospital	Keratinocytes	18 December 1997
Private		
Bone Therapeutics	Bone marrow stem cells	30 June 2010
Cardio 3 BioSciences	Cardiac progenitor cells	1 April 2011
TiGenix	Autologous chondrocytes^a^	1 December 2009

^a^ChondroCelect^®^, the first EC authorized ATMP on the EU market

#### Outcome of the ATMP regulation

In 1999, initiatives like LIFE (Living Implants from Engineering, USA) promised to be able to tissue engineer human organs (e.g. hearts) within 10 years (Zandonella 2003). In 2000, the Scientific Committee on Medicinal Products and Medical Devices warned that the absence of a specific regulatory mechanism would hamper the *imminence of commercialization* of hTEPs (European Union 2001c). According to the 2003 DG JRC-IPTS study (Bock et al. 2003), sophisticated and novel hTEPs (e.g. tissue-engineered intervertebral discs, larger bone substitutes and heart valves) would become available *in the foreseeable future* (Table 1—5.1). Today, more than a decade later, it seems that at least some of these expectations were glossed over. In a 2003 Nature paper, LIFEs chief visionary conceded that LIFEs 10-year timescale was unrealistic (Table 1—5.2; Zandonella 2003). “We were trying to capture the attention of the public,” he admitted. Four years after its implementation, the net outcome of the ATMP Regulation is very disappointing. Seventy requests for ATMP-classification submitted to EMA’s CAT resulted in eight marketing authorization applications (EMA/CAT 2012b) from which only one was granted ATMP market authorization by the Commission: ChondroCelect^®^, characterized autologous chondrocytes (EMA 2009). On 20 July 2012, on its fourth attempt, a second ATMP—Glybera^®^, a gene therapy product for the treatment of lipoprotein lipase deficiency, obtained a positive opinion that recommends marketing authorization (EMA/CAT 2012b). The creation of a hTEP hype without subsequent delivery did, however, create a playing field for charlatans offering unsafe therapies, such as unproven stem cell therapies practiced outside the standard clinical trial network, threatening the cause of legitimate clinical investigation (Daley 2012).

#### The hospital exemption (HE) rule

It seems mind-blowing that public hospitals and laboratories will be encouraged—to put it mildly—to squeeze their established “advanced” therapies through the ATMP funnel where numerous companies have failed. This view was shared by EMA (Table 1—10.4) and by the EC, which introduced the HE rule (Article 28 of the ATMP Regulation) to allow hospitals to provide non-routine ATMPs for an individual patient in the transitional period or in case of high-unmet medical need because there is no authorized ATMP alternative available. The exemption applies to any ATMP, prepared on a non-routine basis according to specific quality standards, and used within the same Member State in a hospital under the professional responsibility of a medical practitioner, in order to comply with an individual medical prescription for a custom-made product for an individual patient. Products that meet the ATMP definition, but fall under the scope of the HE are exempted from the obligation to be authorized via the centralized procedure. Member States are requested to lay down rules for authorizing these products by the NCA whilst at the same time ensuring that relevant Community rules related to quality and safety are not undermined. Traceability, quality and pharmacovigilance standards for ATMPs under the HE should be *equivalent* to requirements for a centralized marketing authorization. Not surprisingly, this rather subjective description gave rise to serious discussions between and within national cell and tissue banking communities and NCAs. In The Netherlands, a HE will only be granted for maximum 10 applications per year. In the UK, the MHRA considers that it is not feasible to provide a simple numerical formula that would delineate the boundary between routine and non-routine production. The UK “specials” scheme, set up under the derogation permitted in Article 5 (1) of the medicinal products Directive 2001/83/EC (European Union 2001a), permits doctors and certain other prescribers to commission an unlicensed relevant medicinal product to meet the special needs of individual patients. In principle this scheme is available for ATMPs as for any other category of medicinal product. There is a “special needs test,” interpreted to mean the absence of a pharmaceutically equivalent and available licensed product. In other words, unlicensed ATMPs could be authorized when no equivalent licensed products are available (Lowdell 2012). The Belgian legislation for applying HE is still under development (Mush 2012). Dossier requirements for HE, including information on production process and environment and safety and efficacy data, are being developed. It is however clear that in the end in Belgium—as is already the case in several other Member States—GMP principles, which imply major investment in upgrading manufacturing facilities, will generally apply and that only very slight deviations will be tolerated and this on a case-by-case basis. It is also clear that different interpretation of the HE by NCAs does not go in the sense of European harmonization. In addition, van Wilder argues that there is evidence that the HE rule is a threat to the aim of the ATMP Regulation of guaranteeing the highest level of health protection for patients (Van Wilder 2012). We tend to agree, as it is very hard to obtain the experience and training necessary to guarantee the best quality of work when production is only sporadic (e.g. less than 10 applications per year). Finally, it is rather strange that an *exemption* rule will actually need to accommodate for the *majority* of ATMPs.

#### Public cell and tissue banks are up against the wall

The last decade, the HCT/P transplantation field made considerable efforts to conform to the national transpositions of the EUCTDs. Some public cell and tissue banks were not able to comply and threw in the towel. Others, which indeed had a rather nonchalant attitude towards quality and safety, were no longer licensed by the NCAs. In Belgium, for example, the number of authorized bone banks was reduced by a third (FAMHP 2012b). Some survivors of this partition are now confronted with the ATMP Regulation, which imposes without distinction and without strong scientific support (e.g. quality and safety under the EUCTDs was not evaluated) another layer of expensive pharmaceutical industry standards (e.g. GMP compliance and marketing authorization), even when their products will never reach the EU market. These requirements were designed for and in collaboration with pharmaceutical companies, which typically produce large batches of drugs for application in many patients. Where EU centralized marketing authorization may provide an incentive for companies, hospitals or academic centers do certainly not aim at holding such an authorization. Pharmaceutical industry standards are not compatible with niche applications where economies of scale don’t apply, or with “à la carte” and often single patient procedures with limited time lines (Apperley 2012). This applies to both SMEs and public tissue establishments. GMP facilities, for example are only profitable when producing large batches of products. Most hospital cell and tissue banks have a vested interest in providing meaningful, reasonably priced, often tailor-made treatments to (niche) patients, and this on a non-industrial scale. In addition, in some Member States like Belgium, these banks are not allowed to make profits. They can thus focus on doing the things that yield better health outcomes without having to maximize profits in the process. But, as mentioned before, the competition of established public actors, which provided “hidden” ATMPs at minimum price—i.e. without (overzealous) pharmaceutical industry requirements and without margin of profit, was perceived as unjust and troublesome by the emerging tissue engineering industry. As predicted in the ATMP IA report, the trend of concentration is likely to continue (Table 1—6.2) as many hospitals and tissue banks may abandon their ATMP efforts in the near future. To safeguard some life-saving therapies, and because medicine won’t stop on its way, the field might evolve to circumvent legislation and find refuge under the umbrella of the “Declaration of Helsinki” or the “single surgical procedure” rule (e.g. peri-operative processing of cells; Table 1—10.13). The single surgical procedure is indeed an easier alternative to pursue medical advances, but it lacks some of the quality and safety aspects and the oversight of HCT/Ps produced and delivered by cell and tissue banks.

### The impact of the EU HCT/P legislation on Member States’ health care systems

#### Indirect impact

The costs of ATMPs are much higher compared to established conventional treatments, as developers need to compensate their investments. Reimbursement of these costs by the government is a contentious issue, yet it is crucial for the survival of ATMP producers and for making ATMPs available to those in need (Table 1—10.5). Access to hTEPs depends on more than just product availability. Treatments must be affordable for patients and healthcare systems for them to be applied in the long run. The second DG JRC-IPTS study report (Bock et al. 2005) states “reimbursement policies are particularly significant” and “currently, hTEPs are much more expensive than conventional treatment options and cost-effectiveness data are scarce” (Table 1—6.5). The report also states “downstream players such as doctors, patients and insurers might face higher product prices as companies seek to their increased compliance (to national and EU standards) costs” (Table 1—6.4). However, when assessing the impact of the ATMP regulation on public expenditures, the EC chose to focus on the extra costs of implementing and maintaining the legislation (Table 1—9.11). Almost anecdotally, the IA report does mention, “lastly, there may also be a potential *indirect impact* on public expenditure through pricing and reimbursement of advanced therapy products,” but—conveniently and rightfully—points out that “the pricing and reimbursement aspect falls under the responsibility of Member States” (Table 1—9.12). Pricing and reimbursement issues will soon become critical as production to GMP and centralized or national (when HE applies) marketing authorization requirements will inevitably multiply the price of the 22 (Table 2) “uncloaked” ATMPs. In Belgium, health care insurance is part of a social security system. Medical costs are reimbursed by a health insurance fund and the government fixes reimbursement rates. Reimbursement rates of “conventional” HCT/Ps are published in a ministerial decree (FAMHP 2009) that also fixes the price of lyophilization and WHO-approved prion- and virus-inactivation techniques. This price system was installed to cover the real procurement and processing costs and to leave no room for unreasonable profits. In 2011, the Belgian stock market listed bio-medical company that produces the first and only EC authorized ATMP was granted national reimbursement for their product (ChondroCelect^®^ for the treatment of symptomatic knee cartilage lesions; TiGenix 2011). Not surprisingly, the reimbursement price is nearly ten times the price of non-ATMP autologous chondrocyte cultures and—due to these high costs—reimbursement is restricted to patients younger than 50 years. The reimbursement of ChondroCelect^®^ to only a part of the needy Belgian patients is in conflict with the equal access to health care, which is one of the leitmotivs of the Belgian public healthcare system. It indicates that the increased costs of pharmaceutical production and marketing requirements are *indirectly* hampering the access to cellular therapies. Who will then have access to future cell therapies, “self-paying downstream players” to use EC wording, or the “happy few” to use everyday wording? Ideally, every needy patient should have access to HCT/Ps, but in the light of the (unnecessary) high cost of ATMPs and an ever more rising public health spending and the current economic crisis, allocation criteria will need to be established. Should young people with complex fractures have access to autologous chondrocytes or elderly persons with arthritis? Anyway, is restricted allocation a viable option for SMEs already targeting a niche market? For industry the logical way ahead would be to lobby for harmonized reimbursement of all ATMPs (for their authorized clinical indications) that make it to the EU market (Table 1—10.12). This would fit well in the Lisbon Strategy, which works towards the achievement of equity and solidarity through optimized social protection systems. Some EU initiatives are already touching on the pricing and reimbursement of medical devices, such as the upcoming Cross Border Healthcare Directive, which provides for a basis for cross-border health technology assessment that will obviously have an influence on pricing and reimbursement (Vollebregt 2011). So, even though the EU has no competence to legislate directly on the subject of pricing and reimbursement of healthcare, it will find its ways to exert indirect influence where possible. The industry stands to gain a lot from harmonized rules and procedures with respect to pricing and reimbursement.

Because, once an ATMP has obtained marketing authorization, the pressure on companies and authorities to provide reimbursement becomes harmfully high, it has been suggested that industry and reimbursement authorities should decide which ATMPs will warrant future reimbursement (for every needy patient) and this prior to their development (Pirnay et al. 2012a).

#### Direct impact

For many years therapies involving tissue engineering (e.g. cell expansion) have been provided by hospitals. These established therapies are often lifesaving [e.g. tumor vaccines (Palucka and Banchereau 2011) and keratinocytes for severely burnt patients (De Corte et al. 2012)] and their development was done in good faith and with good intensions (Apperley 2012) and was characterised by a gradual emergence of efficacy (Table 1—10.3). In addition, if they are still authorized today, they can most likely present a proven track record of quality and safety enhancement under the EUCTDs. As mentioned before, some public hospitals and laboratories are bound to abandon the production of these established therapies. In addition, some commercial products that were already authorized by national authorization systems might also be taken away from patients (Table 1—11.2). It is up to the pharmaceutical industry to fill the imminent gap caused by the ATMP Regulation they asked for and warmly welcomed, in the name of patient safety (Table 1—10.6, 14). But, as it costs an estimated $1.8 billion to bring a new drug to the market (Paul et al. 2010), pharmaceutical companies tend to concentrate on potential best sellers that can be sold to millions of people (European Union 2001b). The global cell therapy product revenue (16 leading commercial products) for 2011 was estimated to be $0.73 billion (Buckler 2012). All patients hope lie thus with SMEs, which have the potential to pursue niche markets and after all they also welcomed the new ATMP Regulation, including the centralised marketing authorization procedure approach (Table 1—8.4, 10.10,16). Unfortunately, as mentioned before, only two SMEs successfully completed the certification procedure, resulting in two ATMPs on the EU single market.

Today, some “innovative” therapies are thus exclusively provided by the public sector. So, if this sector is not able (or willing) to implement requirements for drugs or fail to get (central or national) marketing authorizations, some valuable established therapies will be made unavailable in several Member States.

In addition, most of the therapies that are in development in academia and SME’s have not yet reached the step of clinical trials, which means complicated approval under the new Regulation. Ultimately, patients will suffer or even die, unjustly. The ATMP Regulation will thus have a *direct* adverse impact on MDs ability to treat patients.

#### Examples

Today in Belgium, “EUCTD compliant” keratinocyte cultures are applied on severely burnt patients at a price of €5.92 per cm^2^ (the Belgian reimbursement price; FAMHP 2009). If Belgian keratinocyte banks were led to abandon their keratinocyte graft production (due to financial reasons and/or reservations on principle), Belgian burn wound centers would need to buy “ATMP compliant” keratinocyte cultures on the EU market. Alternatives are MySkin^®^, produced by Altrika Ltd in the UK (authorized under the “specials” scheme, which makes export to Belgium uncertain) and EpiCel^®^, manufactured in the US by Genzyme (no EU marketing authorization up till now). Prices vary from €13 to 20 per cm^2^. The outsourcing of keratinocyte production will lead to a delay and a loss of flexibility in therapy, mainly due to the forward and backward cross boarder transports of respectively the starting material (a skin biopsy) and the resulting graft. In addition, if the Belgian reimbursement price is not increased proportionately, the hospitals will need to reserve this therapy, if anything, for young children. The application of keratinocytes exclusively on patients with private health insurances is no option for most hospitals.

In November 2011, EMA’s CAT classified bone marrow mononuclear cells, intended for the treatment of ischemic syndromes, as ATMPs instead of as cellular transplantation. Cuende et al. warned that this will have a very negative impact on EU public health services and on patients who will have to wait longer and pay more for their treatments (Cuende et al. 2012).

Already in 2007, Trommelmans et al. suggested that the development and application of hTEPs might influence European healthcare in two respects: the conditions for the application and reimbursement of hTEPs, and the allocation of hTEPs to individuals (Trommelmans et al. 2007a).

### Academia paralyzed

Academia and public institutions did not defend in full their interests and those of the most important stakeholders, the patients. It is often suggested that this is mainly because they are not sufficiently resourced, experienced and organized to influence policy. But, we feel that the public sector should also search in its own conscience. In contrast to industry, academia and public institutions were unable to provide sufficient data on the scope and extent of their tissue (engineering) activities, which has certainly played a role in the underrepresentation of this sector in targeted consultations. The in-house science service of the EC (DG JRC-IPTS) should at least have observed this bias. The underrepresentation of academia and the public sector in public consultations can be assigned to inexperience, unawareness and a lack of time. Because they can’t (afford to) rely on dedicated regulatory affairs officers, MDs with a full-time job have to defend their interests and these of academia and public institutions. It must also be said that some renowned academic experts simply didn’t bother to participate in the policy shaping process. They underestimated the scope and the impact of EU legislation and thought they would be able to negotiate a solution to eventual adverse impacts, ad hoc and at a national level—as they were accustomed to do. But, even now that the negative impact of the overzealous requirements on the public HCT/P sector and patients are materializing, academia—which has power to influence policy—hardly reacts. This may be a consequence of the discord that exists within academia and the deepening ties between academia and industry. Over the last few decades, universities have shifted towards the ‘entrepreneurial university’ model (Etzkowitz 2003) that refers to the increasing tendency to run the university as a quasi-business with an emphasis on contract research, a very active patent and licensing policy and the establishment of spin-offs in the fields of innovative drug design, materials development, translational medicine and medical technology and devices. Some university professors are even member of boards of directors of spin off companies that develop innovative HCT/Ps. They were mostly unaware of the fact that business oriented HCT/P regulation would also apply to their university spin offs.

### Industry’s nature

It is not surprising nor chocking that industry lobbied for business oriented legislation. According to the market economy paradigm, the social responsibility of business is to increase its profits, not to relax the conditions of profit-maximization on behalf of the wider interests of society (Friedman 1970). Company executives have to take into account the interests of their employees, shareholders and the long-term interests of the company. As a consequence, companies do not always see service to the general public as a key priority. Since long, public cell and tissue banks and laboratories that manufacture hTEPs are perceived by industry as unjust competitors, and companies are known to eliminate competitors (e.g. by forcing them into bankruptcy or preventing new firms from entering the industry). But, as in the fable about a scorpion asking a frog to carry him across a river, industry might well have compromised its own stakes. In a crucial phase in the development of regenerative medicine (midway across the river) industry (the scorpion) lobbied for industry standards to be imposed on the public sector (stung the frog), dooming both of them. When asked why, the scorpion pointed out that this is its nature.

### The social responsibility of policymakers

Political authorities, much more than private companies, have a social responsibility to promote public health in the most efficient way they can. Unfortunately, policymakers are sometimes unaware of how changes to policy are taking effect or who is behind them, an issue, which may be particularly pertinent in the EU, which is a complex political system with multiple points of access and into which business interests are historically highly integrated (Smith et al. 2010a). Who undertakes IAs, on whose behalf, who provides the required resources including the data, who decides which stakeholders are involved or excluded, who influences methodology and who validates results (Smith et al. 2010a)? Nevertheless, several reports of studies commissioned by the EC *did* mention potential adverse impacts of the HCT/P legislation. The first IPTS study report mentioned allocation and cost-effectiveness issues such as the development of tissue-engineered skin products for the “self-payer” patients segment (e.g. aesthetic surgery) and a lack of strong evidence for superiority and cost-effectiveness of tissue engineering treatments over the conventional treatment (Table 1—5.6-7). The ATMP IA predicted the competition between tissue engineering companies and hospitals and tissue banks and pricing and reimbursement issues. Unfortunately, these adverse impacts were swept under the carpet, as they would respectively “remain limited in the short to medium term” and “fall under the responsibility of Member States.” Policymakers should not be allowed to hide behind cost-based (economic) options to protect the interests of private companies. They should assume their social responsibility. If it can be shown that in certain cases public tissue banks and laboratories are the best solution to promote overall health benefits, compared to eventual commercial alternatives, then there is a public moral requirement to do so. The need for regulation of HCT/Ps cannot be denied, but the regulatory framework must be *proportionate*, tailored to the actual players in the field and enabling the development and timely and horizontal access to conventional and innovative therapies (Table 1—8.2). EMA recently admitted that the complexity of the current legislation prevents providers bringing their therapies to the market due to a lack of resources to comply with the regulatory standards and wants to foster the development of advanced therapy by strengthening the dialogue with the stakeholders and the help given to them (EMA/CAT 2010). The clinical routine in the hospitals performing the cellular therapies must be taken into account by the revision of the legislation (Klumb 2011). Meanwhile, irreversible damage is being done as some hospitals and SMEs are abandoning their efforts due to insurmountable hurdles or fundamental objections. The ATMP Regulation should thus *urgently* be revised to focus on delivering affordable therapies to all who are in need of them and this without necessarily going to the market. In particular, we feel that tailor-made and/or niche ATMPs, provided by public institutions and SMEs alike—a level playing field is indeed appropriate—should not face requirements that go beyond the accreditation system and the quality and safety standards laid down in the EUCTDs and this for all aspects of their existence, from donation to distribution. Unless the EUCTDs are *proven* to be insufficient to ensure patient safety (not market access), these non-commercial ATMPs should be kept outside of the scope of the Medicinal Product Regulation. The EC could, for instance, publish an interpretative document on “placing on the market of ATMPs” to achieve this. Moreover, it seems unlikely that unnecessary stringent regulation will ultimately benefit the emerging EU tissue engineering market. To maximize on profit, but also to flee restrictive US and EU regulation, “big pharma” and biotech companies increasingly outsource drug discovery, development and manufacturing to off shore countries like India and China, which are not characterized by a predominance of risk-averse regulatory environments.

### Health care should be disconnected from market rationality

In the EU, health care systems have traditionally been patient-driven and based on the principles of human dignity, equity (of access), quality (highest possible) and solidarity (of financing). Social health care systems are under increasing pressure from such factors as aging populations, expensive innovative treatments (e.g. ATMPs), rising public expectations (e.g. the stem cell hype), intensified fiscal pressures generated by the current global financial and economic crisis, and cross-border patient flows (Flear 2009). In addition, the aims of DG Sanco to protect public health are increasingly subdued to DG Enterprise’s aims to promote trade. In this context it is not surprising that the recent EU HCT/P regulatory framework will deliberately create a global market for uniform HCT/Ps in which public cell and tissue banks supply human cells and tissues to corporate firms. In a two-tier approach the EU first set the bar just high enough (the EUCTDs) to ensure that large public banks would still be able and willing to provide high quality cells and tissues to an anticipated tissue engineering industry. In a second phase (the ATMP Regulation) the EU ensured that the role of these public banks would be exactly limited to that. They would not (unjustly) compete with tissue engineering companies. There are striking parallels with the food sector. The rising liberalization of agro-industrial markets was also steered by technological advances and the introduction of a EU regulatory framework. Small food producers, unable or not willing to go along with technological advances and new ideologies in marketing, are suffering under the new product safety regulations. Established tasty local products are gradually replaced by uniform insipid global brands, with a perception of high quality and safety. Mark Flear (2009) analyzed the discourse of the relaunched Lisbon Strategy and the techniques of the OMC on health care and observed a growing linkage of equity and solidarity with optimization in the context of “modernization,” which serves to place health care within a neoliberal frame. Neoliberalism seeks to organize policies in both market and non-market spheres by extending and disseminating market rationality and economic behavior (competition, privatization, profit maximization, globalization and contracts) into nonmarket spheres including health care, which are thereby subordinated. For example, in the newly framed HCT/P transplantation field altruistic cell and tissue donations are subjected to market forces and standards. But, how did the EU manage to bypass subsidiarity of Member States regarding health care systems? As explained before, the Amsterdam Treaty allowed the EU to implement industry quality and safety requirements to protect the EU citizen’s health and safety. But, Member States’ health care systems were still off limits for the EU. With the Lisbon Strategy and in particular the OMC the EU could go one step further. Europe’s supranational community’s main focus has always been on economic progress and industrial innovation. Basically, neoliberalism aimed at accumulating wealth in a few hands with the argument that it would promote investment, thereby creating more jobs and more prosperity for all. The choice for neoliberalism was thus logical; who does not want economic growth and jobs? But, the current global financial crisis has revealed that instead of creating jobs, speculative investments predominantly fed an ephemeral prosperity that could be wiped out in a short time period (Beder 2009). Experts increasingly caution that the liberalization of the EU market might leave behind the EU’s weakest economies. As is the case for the overarching EU market, the newly formed commercial health care market might also leave behind its weakest links, less well-off patients.

The model neoliberal citizen is one who strategizes for her- or himself among various social, political and economic options, nor one who strives with others to alter or organize these options. According to Le Grand (2003) it does seem as though there is a convincing case for the user to have a measure of power over public service provision. Liberal egalitarians support redistribution required for universal health care and think of individual fates as tied together, and of benefits of social cooperation as to be shared out among participants (Risse 2005). Reflection is required on what is more important, communal or individual welfare, the consumer citizen or the patient-citizen? With the establishment, in 1951, of the European Coal and Steel Community, the EU started off as a purely economic structure and, in essence, it still is today. We feel, however, that health care should be disconnected from the cynicism of free market economy.

## Conclusions

### In the name of safety

In the late nineties, at the peak of the hTEP hype, industry incited EU policymakers to create a European regulatory environment that would facilitate the emergence of a strong internal market for hTEPs. Officially, industry representatives and policymakers emphasized that EU HCT/P legislation was urgently needed to provide protection for public health. The reason for this is that the EU is exclusively competent for economic aspects such as the internal market, monetary policy and biological resources, but there can be no question of harmonization for certain social matters such as public health. In other words, public services such as health care do not subordinate to the internal EU market. For “common safety concerns in public health,” however, both the EU and Member States are authorized, but Member States can only act if the EU does not act or decides not to act. This means that if the EC would want to regulate matters that touch public health, such as the HCT/P transplantation field, the only gateway would be “common safety concerns in public health” in an area in which application of existing Community legislation and additional national measures have proven insufficient. The mediatized safety and ethical scandals involving human cell and tissue transplants, which questioned the oversight of the HCT/P transplantation field in the late nineties, presented the EC with an ideal opportunity to issue HCT/P legislation.

### Industry’s influence

Naturally, different stakeholders had different interests, resources, values and aims in the HCT/P field. The considerable influence of industry on all levels of the EU policymaking process (studies, consultations and IA process) was however decisive. Efficient industry lobbying lead to asymmetry (towards industry) in studies (e.g. the 2003 IPTS study wrongfully concluded that hospitals did not yet produce any hTEPs), consultations and IA, and ultimately to the provision of distorted information to the EU institutions about the possible economic, social or environmental impact of draft legislative proposals. Industry’s point of view predominated because debates are made following their rules, their tools and language and practices (lobbying), which are not the academic’s ones.

In summary, industry successfully lobbied to:

- Create the new entity of tissue establishment as more wide-ranging than conventional notions of cell and tissue banks. As such, in most Member States, the procurement of human cells and tissues falls within the purview of industry.
- Include ATMPs, a subset of HCT/Ps, within the medicinal products regulatory framework. As a consequence they must comply with requirements for pharmaceutical drugs (e.g. GMP compliant production and marketing authorization).
- Obtain a level playing field. The creation of a HE backdoor could not be prevented, but industry managed to keep it so tight that hospitals are not likely to squeeze in.
- Prevent the distinction between autologous and allogeneic HCT/P applications.
- Exclude contentious social and ethical issues, such as the commercialization of human bodily material, from the HCT/P legislation.
- Blur the distinctions between non-profit and profit-making activities of tissue establishments. The profit that can be made from the processing of human cells and tissues is not limited.

### Impact on the HCT/P transplantation field

The main motive for EU HCT/P legislation was to facilitate the growth of an emerging human tissue engineering industry. It is thus not surprising that instead of dealing with controversial market-driven practices that plague the HCT/P transplantation field, it actually creates a favorable environment for legal excessive profit making activities (LEPRAs) by facilitating the development of a single HCT/P market with for-profit tissue establishments and without limiting the profit that can be made on the processing of human cells and tissues. Pharmaceutical industry standards were introduced and ethical issues were left to be dealt with by the Member States, insofar possible. This resulted in a risk-averse and patched EU regulatory environment from which nobody—patients, public and private tissue establishments alike—stands to benefit. Industry’s access to primary material is not guaranteed and the HE rule produces disorder and uncertainty amongst NCAs and tissue establishments. The HCT/P legislation was requested and welcomed by the pharmaceutical and biotechnology industry, but now it turns out that “big pharma” is not really interested and that SMEs are struggling to get their products through the medicinal product accreditation funnel. Public cell and tissue banks, the actual suppliers of “advanced therapies” are up against a wall of industry requirements. The only players that will actually gain from the new HCT/P regulatory environment are those supplying GMP equipment and consumables, those offering custom development and (GMP) biopharmaceutical manufacturing services (Table 1—6.3) and pharmaceutical companies in search of priceless research tissues. The EU HCT/P legislation, which was developed under the pretext of improving public health safety, will not only *indirectly* (pricing and reimbursement), but also *directly* (the imminent loss of meaningful therapies) impact Member States’ social health systems. The ATMP IA concluded that the proposed regulatory options would only have an *indirect* impact on Member States’ health care systems. A *direct* impact would have meant a breach of the subsidiarity principle and the ATMP Regulation would probably not have been adopted in its actual form. Because medicine won’t stop on its way, the field might evolve to develop easier, but less safe alternatives to the HCT/Ps that are currently produced by public cell and tissue banks under the EUCTDs, but will become unreachable under the ATMPs Regulation. For example, cells and tissues can be processed peri-operatively and applied in a single surgical procedure, thus evading compliance to the ATMP Regulation as well as the EUCTDs. The most concerned stakeholder, the EU patient, which the EC somewhat lost out of sight, will ultimately be the victim.

### Towards a workable solution

EU policymakers should urgently assume their social responsibilities and safeguard the horizontal and timely access to affordable, safe, efficient, and ethically sound advanced therapies. For this they could:

- Put forward a clear ethical position that overcomes commercialization issues. For instance, the commercialization of human bodily material could be tolerated when tissue establishments act in good faith and produce HCT/Ps for use in meaningful therapies. The good faith of tissue establishments could be reflected in a HCT/P cost price that only relates to the added technological process, and this in a reasonable manner.
- Keep tailor-made and niche ATMPs outside of the scope of the Medicinal Product Regulation (unless the EUCTDs are *proven* to be insufficient to ensure patient safety). To achieve this in a timely manner, the EC could publish an interpretative document on “placing on the market of ATMPs.”
